# Gene expression of *Porphyromonas gingivalis* ATCC 33277 when growing in an *in vitro* multispecies biofilm

**DOI:** 10.1371/journal.pone.0221234

**Published:** 2019-08-22

**Authors:** Patricia Romero-Lastra, María C. Sánchez, Arancha Llama-Palacios, Elena Figuero, David Herrera, Mariano Sanz

**Affiliations:** 1 Laboratory of Dental Research, Faculty of Odontology, Complutense University of Madrid, Madrid, Spain; 2 ETEP Research Group, Faculty of Odontology, Complutense University of Madrid, Madrid, Spain; East Carolina University Brody School of Medicine, UNITED STATES

## Abstract

**Background and objective:**

*Porphyromonas gingivalis*, an oral microorganism residing in the subgingival biofilm, may exert diverse pathogenicity depending on the presence of specific virulence factors, but its gene expression has not been completely established. This investigation aims to compare the transcriptomic profile of this pathogen when growing within an *in vitro* multispecies biofilm or in a planktonic state.

**Materials and methods:**

*P*. *gingivalis* ATCC 33277 was grown in anaerobiosis within multi-well culture plates at 37°C under two conditions: (a) planktonic samples (no hydroxyapatite discs) or (b) within a multispecies-biofilm containing *Streptococcus oralis*, *Actinomyces naeslundii*, *Veillonella parvula*, *Fusobacterium nucleatum* and *Aggregatibacter actinomycetemcomitans* deposited on hydroxyapatite discs. Scanning Electron Microscopy (SEM) and Confocal Laser Scanning Microscopy (CLSM) combined with Fluorescence *In Situ* Hybridization (FISH) were used to verify the formation of the biofilm and the presence of *P*. *gingivali*s. Total RNA was extracted from both the multispecies biofilm and planktonic samples, then purified and, with the use of a microarray, its differential gene expression was analyzed. A linear model was used for determining the differentially expressed genes using a filtering criterion of two-fold change (up or down) and a significance p-value of <0.05. Differential expression was confirmed by Reverse Transcription-quantitative Polymerase Chain Reaction (RT-qPCR).

**Results:**

SEM verified the development of the multispecies biofilm and FISH confirmed the incorporation of *P*. *gingivalis*. The microarray demonstrated that, when growing within the multispecies biofilm, 19.1% of *P*. *gingivalis* genes were significantly and differentially expressed (165 genes were up-regulated and 200 down-regulated), compared with planktonic growth. These genes were mainly involved in functions related to the oxidative stress, cell envelope, transposons and metabolism. The results of the microarray were confirmed by RT-qPCR.

**Conclusion:**

Significant transcriptional changes occurred in *P*. *gingivalis* when growing in a multispecies biofilm compared to planktonic state.

## Introduction

The oral cavity is a unique ecological environment colonized by more than 500 bacterial species [1–3]. These bacteria are part of the oral microbiome and may be floating freely or within structured bacterial communities being part of complex biofilms, which provide bacteria protection against shear forces and host immune responses [4–6]. If these biofilms are not allowed to grow and mature, mainly through effective oral hygiene practices, these stable communities develop immune tolerance and may remain in symbiosis with the oral tissues. However, if they increase in mass or there are relevant changes in the local environment that favors the growth of pathobionts (dysbiosis), the immunological tolerance will be surpassed leading to inflammation [7, 8]. Among these pathobionts, *Porphyromonas gingivalis* has shown the expression of virulence factors to evade the host responses and to favor its colonization and spread within the tissues [9, 10].

Several studies have shown that when *P*. *gingivalis* grows within a biofilm, specific genes will become differentially regulated [11–14]. These genes may be relevant in promoting phenotypic adaptations of this pathogen, what may facilitate its infective potential [15–17], mostly by evading the immune response and promoting non-resolving chronic inflammation resulting in soft and hard tissue destruction, which are the key pathological features of periodontitis [5, 9, 18–20]. There is, however, scarce transcriptomic information of *P*. *gingivalis* and most available knowledge on the gene expression comes from *in vitro* monospecies biofilm models [21, 22]. Our research group has recently reported significant differences of gene expression when *P*. *gingivalis* was growing within a monospecies biofilm, mainly in those genes related to cell envelope, transport, outer membrane proteins, transposases and oxidative stress genes [14]. Furthermore, significant differences were encountered in those genes related to metabolism, adhesion, invasion, virulence and quorum sensing, when a growing monospecies biofilm was in the presence of planktonic *P*. *gingivalis* [23]. However, within the oral cavity, symbionts and pathobionts colonize as multispecies biofilms [7, 19, 24–26], and these bacteria will be faced with diverse DNA exchanges (horizontal gene transfer) and to multiple stressors, but only those bacteria with expressed genes that will enable them to colonize or resist host defenses, will be able to survive and predominate [2, 3, 27–30]. It was, therefore, the purpose of this *in vitro* study to compare the gene expression of *P*. *gingivalis* when growing within a multispecies oral biofilm with its growth in planktonic conditions.

## Material and methods

### Bacterial strains and culture conditions

Methodology for developing the multispecies biofilm was similar to that previously reported from our research group [14, 23]. Briefly, reference strains of *P*. *gingivalis* ATCC 33277, *Streptococcus oralis* CECT 907T, *Actinomyces naeslundii* ATCC 19039, *Veillonella parvula* NCTC 11810, *Fusobacterium nucleatum* DMSZ 20482 and *Aggregatibacter actinomycetemcomitans* DSMZ 8324 were used. Each bacterial strain was grown on blood agar plates (blood agar Oxoid no. 2; Oxoid, Basingstoke, UK), supplemented with 5% (v/v) sterile horse blood (Oxoid), 5.0 mg/L hemin (Sigma, St Louis, MO, USA) and 1.0 mg/L menadione (Merck, Darmstadt, Germany) under anaerobic conditions (10% H_2_, 10% CO_2_ and 80% N_2_) at 37°C for 24–72 h.

### Experimental assays

**Fig 1:**
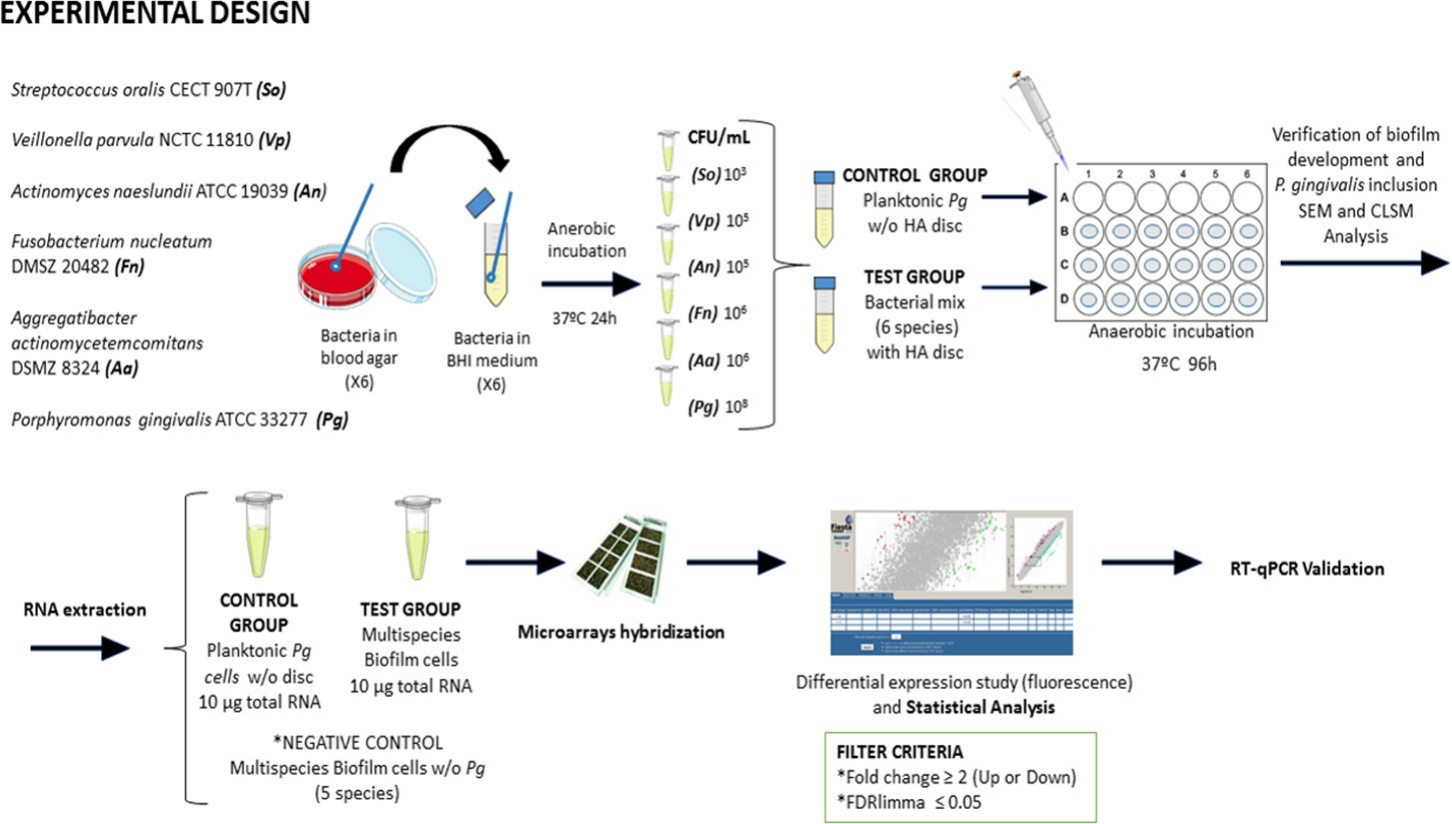
Overview of experimental design. *P*. *gingivalis* ATCC 33277 was incubated in anaerobiosis at 37°C for 96 h and grown under planktonic conditions (control group) and within a multispecies-biofilm on hydroxyapatite (HA) discs, also containing the other described five bacterial species (test group). Scanning Electron Microscopy (SEM) and Confocal Laser Scanning Microscopy (CLSM) were used to verify the multispecies-biofilm development and the presence of *P*. *gingivali*s in it. Total RNA was extracted, purified and the differential gene expression was analyzed by microarray [Agilent *P*. *gingivalis* Oligo Microarrays 8x15K (074976)] with a filtering criterion of two-fold change (up or down) and significance p-value <0.05. Differential expression was confirmed by Reverse Transcription-quantitative Polymerase Chain Reaction (RT-qPCR). (Images for Fig 1 were taken from https://smart.servier.com/ under a creative commons license).

Fig 1 depicts the experimental design of this investigation. Planktonic cultures of each reference strain were grown anaerobically at 37°C for 24 h in a protein-rich medium containing Brain-Heart Infusion (BHI) (Becton, Dickinson and Company, Franklin Lakes, NJ, USA) supplemented with 2.5 g/L mucin (Oxoid), 1.0 g/L yeast extract (Oxoid), 0.1 g/L cysteine (Sigma), 2.0 g/L sodium bicarbonate (Merck), 5.0 mg/L hemin (Sigma), 1.0 mg/L menadione (Merck) and 0.25% (v/v) glutamic acid (Sigma). By means of spectrophotometry, the late exponential phase growth was verified (optical density at 550 nm). Due to the fact that the bacteria used have different growth rates, and that this biofilm model is static we have adjusted the inocula of the different bacteria as was previously developed and validated by Sánchez *et al*., [31], in order to avoid the overgrowth of certain species and an excessive accumulation of waste products, obtaining by dilution in fresh modified BHI medium the following final concentrations:

- 10^3^ colony forming units (CFUs)/mL for *S*. *oralis*,
- 10^5^ CFUs/mL for *V*. *parvula* and *A*. *naeslundii*,
- 10^6^ CFUs/mL for *F*. *nucleatum* and *A*. *actinomycetemcomitans*,
- 10^8^ CFUs/mL for *P*. *gingivalis*.

Using pre-sterilized polystyrene 24-well tissue culture plates (Greiner Bio-one, Frikenhausen, Germany), two types of growing conditions were developed:

1. The test group (*P*. *gingivalis* growing within a multispecies biofilm). In each well of the plate, 1.5 mL of the mix solution containing the six reference strains, at the above referred concentrations, were deposited together with a sterile ceramic calcium hydroxyapatite discs (HA) [7-mm diameter (standard deviation, SD = 0.2 mm) and 1.8 mm thickness (Clarkson Chromatography Products, Williamsport, PA, USA)];
2. The control group (*P*. *gingivalis* growing planktonically). In each well, a volume of 1.5 mL of pure culture of *P*. *gingivalis* (10^8^ CFUs/mL) was deposited in the absence of discs.

The plates were then incubated in anaerobiosis at 37°C for 96 h. To rule out any possible contamination, a set of wells within the same plate were incubated with only culture medium.

### Monitoring of biofilm development: Scanning electron microscope and confocal laser scanning microscopy

In order to verify the multispecies-biofilm development on discs, Scanning Electron Microscope (SEM) was used. Three HA discs covered with biofilms grown for 96 h were fixed in 4% paraformaldehyde and 2.5% glutaraldehyde for 4h at 4°C, then washed twice in phosphate-buffered saline (PBS) and sterile water (immersion time 10 min) and dehydrated through a series of graded ethanol solutions (50, 60, 70, 80, 90 and 100%; immersion time per series, 10 min). Then, critical point drying and sputter-coating with gold was carried out before analyzing the samples with a scanning electron microscope JSM 6400 (JSM6400; JEOL, Tokyo, Japan) equipped with back-scattered electron detector and with an image resolution of 25 KV.

In addition, in order to ensure the incorporation of *P*. *gingivalis* from planktonic to biofilm, Confocal Laser Scanning Microscopy (CLSM) combined with Fluorescence *In Situ* Hybridization (FISH) was used. Three HA discs covered with multispecies biofilms, grown *in vitro* for 96 h, were incubated for 18 h with 40 μg/ml in hybridization buffer of the 16S rRNA *P*. *gingivalis* ALEXA Fluor 488 probe [5´-3´: CACTGAACTCAAGCCCGGCAGTTTCAA; Life Technologies Invitrogen (Carlsbad, CA, USA)]. Stained biofilms were washed for 15 min in a wash buffer [0.1 M Tris-HCl [pH 7.2], 0.18 M NaCl, 0.05 M EDTA and 0.005% sodium dodecyl sulfate [wt/vol]), and then exposed to 1 μg/mL of DAPI (4',6-Diamidino-2-Phenylindole, Dihydrochloride; Thermo Fisher Technologies, Life Technologies Corporation, Carlsbad, CA, USA) for 5 min. Specimens were then washed 10 min with wash buffer and examined with a fixed-stage Ix83 Olympus inverted microscope coupled to an Olympus FV1200 confocal system (Olympus; Shinjuku, Tokio, Japan). The objective lens was a ×63 water-immersion lens (Olympus) and image stacks were acquired with a z-step size of 0.5 μm thickness (8 bits, 1024x1024 pixels). ALEXA Fluor 488 signals were detected with a PMT detector using a 405 nm laser and an emission range of 647–665 nm, together with DAPI (PMT detector / 552 nm laser / 350–470 nm emission range). Image analysis was performed with Imaris Biteplane software (Belfast, UK).

### Harvesting of planktonic and biofilm cells for gene expression analysis

After 96 h incubation in anaerobiosis at 37°C, samples from both groups, test and control, were harvested and pooled into three biological replicates of each condition for independent hybridization. In the control group, a total of 5 mL were recovered from the wells of the culture plates and sequentially centrifuged to pool them into a single pellet in order to obtain 10 μg of RNA. In the test group, a set of 100 discs per biological replicate were harvested independently in 1 mL of sterile PBS and disaggregated by vortex during 3 min. The disaggregated multispecies biofilms were then pooled in a single sample, to obtain at least 10 μg of total RNA per biological replicate.

Both samples groups (planktonic and multispecies biofilms) were processed in the same manner, centrifuged at 9,000 rpm at 4°C during 5 min and pooled as a single pellet for each biological replicate.

In all cases, an aliquot of each sample was used as quality control. For that, these aliquots were cultured on supplemented blood agar plates under anaerobic conditions at 37°C for two weeks to control for the presence of each intended bacteria and the absence of contamination.

### Total RNA extraction

The three biological replicates of each condition, test and control, were suspended in 1 mL of TRIzol reagent (Ambion, NY, USA) to lysate the cells and to extract the total RNA [TRIzol Plus RNA Purification Kit (Invitrogen)]. Then, 200 μL of cold chloroform was added to separate its hydrophobic and hydrophilic content. The mixtures were then centrifuged at 13,000 rpm for 15 min at 4°C, and the RNA phase collected. Nucleic acids were then precipitated with ~ 500 μL of cold 70% ethanol and centrifuged at 11,300 rpm for 15 seconds at room temperature. Pellets were then suspended in 50 μL of RNase-free water (Roche Diagnostics, Mannheim, Germany). To ensure the absence of any contaminating DNA, DNase I (Ambion, NY, USA) was added to the samples (set of RNase-free DNase; Qiagen, CA, USA) and purified using columns of RNeasy Mini kit (Qiagen) following the manufacturer's protocol.

RNA quantity and quality were measured with Agilent 2100 Bioanalyzer (Agilent Technologies, Santa Clara, CA, USA). An A260/A280 ratio of at least 2.0 was reached for all the samples used in this study.

### cDNA synthesis, labeling and hybridization

The three biological replicates were independently hybridized for transcriptomic comparison of the test and control groups. The fluorescent labeling was performed using SuperScript Indirect cDNA Labelling System (Invitrogen; Carlsbad, CA, USA). Preparation of probes and hybridization were performed following the manufacturer's instructions [One-Color Microarray Based Gene Expression Analysis Manual Ver. 6.5 (Agilent Technologies)]. A slide specific for the strains of *P*. *gingivalis* ATCC 33277 and W83 was used [Agilent *P*. *gingivalis* Oligo Microarrays 8x15K (074976)].

As negative control, two replicates of multispecies biofilm without *P*. *gingivalis* (5 species-biofilm) were prepared and loaded onto the same microarray to rule out any possible gene cross-hybridization from the other bacteria.

### Microarray and data analysis

Images from Cy3 channel were equilibrated and captured with a high-resolution scanner (Agilent) and spots quantified using Feature Extraction software (Agilent) following a similar protocol as in the previous published investigations from our research group [14, 23]. Background correction and normalization of data expression were performed using LIMMA [32–34]. For local background correction and normalization, the methods "normexp" and loess in LIMMA were used, respectively [32]. To ensure consistency and similar distribution across arrays, log-ratio values were scaled using the median-absolute-value as scale estimator [33]. Linear model methods were used for determining differentially expressed genes. Each probe was tested for changes in expression over replicates by using an empirical Bayes moderated t-statistic [33]. To control for false discovery rates, p-values were corrected using the Benjamani and Hochberg method [32, 33]. We selected an expected false discovery rate of less than 5% and a filtering criterium of increase/decrease up to 2-fold differential expression between the two conditions, as used in other similar studies [14, 17]. Expression ratios were expressed as means of the fold changes of the three biological replicates and SD.

The National Center for Biotechnology (Genomics Unit) at Autónoma University of Madrid (Spain) performed the hybridizations and statistical analysis.

The transcriptomic results were inspected manually using different Internet platforms KEGG (Kyoto Encyclopedia of Genes and Genomes) and UniProt.

### Reverse Transcription-quantitative Polymerase Chain Reaction (RT-qPCR)

To validate microarray results, RT-qPCR of selected genes was performed, as described in our previous investigations [14, 23]. Three genes were selected from the up-regulated group and other three from the down-regulated group. Specific primers were designed using the Universal Probe Library Roche software tool, Roche Diagnostics (Table 1). *P*. *gingivalis* 16S rRNA gene was used as a reference gene for normalization of the RT-qPCR.

**Table 1:** Primers used for Reverse Transcription-quantitative Polymerase Chain Reaction (RT-qPCR).

LOCUS NAME	PUTATIVE IDENTIFICATION	PRIMERS FOR RT-qPCR	
PG_2131	OmpA_c-like	Forward Backward	5´-3´: ACACACCCCTCTCGTCTGAG 5´-3´: TCCCTTCCGGATAGCTCTG
PGN_0183 *(FimC)*	Minor component FimC	Forward Backward	5´-3´: CCTTTTCAAGAAAGAACTTGAGGA 5´-3´: GTCGGACTATCGGCTCGTT
PG_1712	Alpha-1,2-mannosidase family protein	Forward Backward	5´-3´: GCTACGAAAGCCGTCCATC 5´-3´: GTACCACTCCCAACCTTTGC
PGN_1058 *(Ftn)*	Ferritin	Forward Backward	5´-3´: GAAATGATCGAGGCTGTCGT 5´-3´: GTCCTGTGATGCCATATCTCC
PGN_0033	Thioredoxin	Forward Backward	5´-3´: CAACATTTGACGGCTTGGTA 5´-3´: CCATGTAGCCCAGAAATCCA
PGN_1208 *(ClpB)*	ClpB protein	Forward Backward	5´-3´: ACAAGGGGCATGTGGTAAAC 5´-3´: AACCGAGGTTCGACGTCAT

The cDNA was generated from 1 μg of total RNA using the High Capacity cDNA Archive Kit (Applied Biosystems, ThermoFisher Scientific) in a 10 μL of final reaction volume. Then qPCR reactions were performed in triplicate by using 5 μL per well of each cDNA, and 3 μL of a mix composed by 0.4 μM of each primer, 5x HOT FIREPol EvaGreen qPCR Mix Plus (ROX), and nuclease-free water, to reach a final volume of 8 μL in 384-well optical plates. PCR reactions were run in an Applied Biosystems ABI PRISM 7900HT machine with Software Defined Storage (SDS) v2.4 and standard protocol from Applied Biosystems (95°C 10 min, 40 cycles of 95°C 15 sec and 60°C 60 sec, and a final standard melting curve dissociation protocol).

All the RT-qPCR measurements were performed in triplicate and the results were analysed with the comparative cycle threshold method (ΔΔCt) [35]. The transcriptional log_2_ ratio from RT-qPCR analysis was plotted against the average log_2_ ratio values obtained by microarray analysis.

## Results

**Fig 2:**
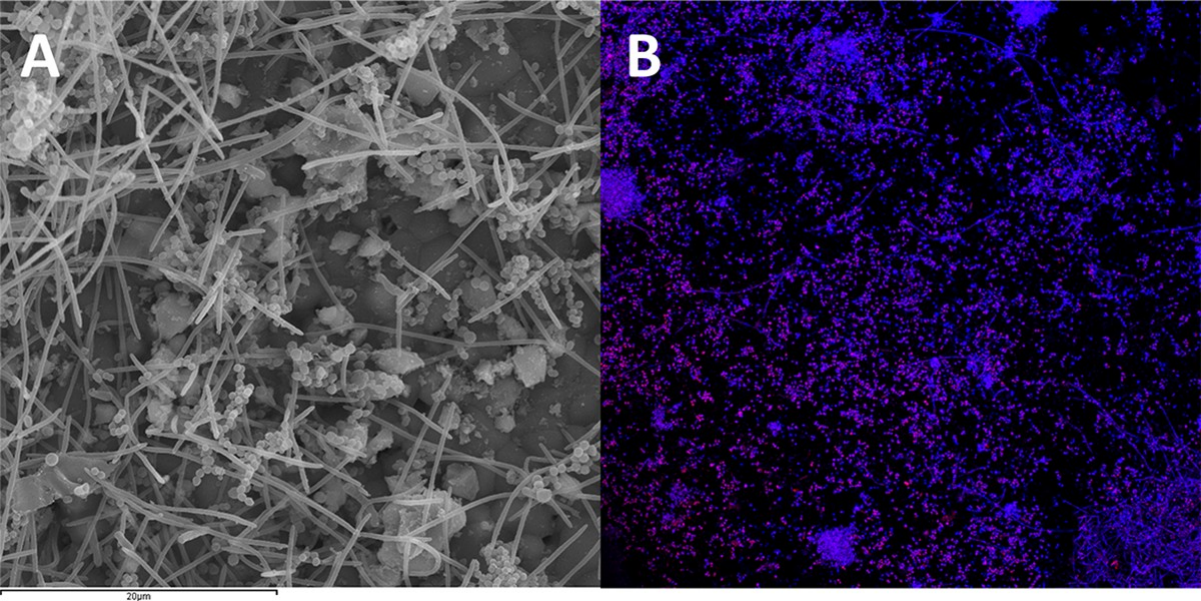
Micrographs representing the multispecies-biofilms after 96 hours of growth. (A) Scanning Electron Microscopy (SEM) depicting the structure of the biofilm. Note the microcolonies organized in clusters with *F*. *nucleatum* connecting them; (B) Confocal Laser Scanning Microscopy combined with Fluorescence *In Situ* Hybridization (CLSM-FISH). The ALEXA Fluor 488 probe detected the 16S rRNA *P*. *gingivalis* (cells in purple) within the multispecies-biofilm (other bacterial species in blue stained with DAPI).

A mature multispecies-biofilm was confirmed by SEM. Fig 2A depicts this multispecies-biofilm, where most of the bacteria were organized in clusters with *F*. *nucleatum* acting as the backbone inter-connecting among the other bacterial morphotype. The CLSM combined with FISH was applied to detect the presence of *P*. *gingivalis* within the mature multispecies-biofilm. Fig 2B depicts the presence of *P*. *gingivalis* highlighted with a fluorescent stain in purple among the rest of bacterial species stained nonspecifically with DAPI in blue.

**Fig 3:**
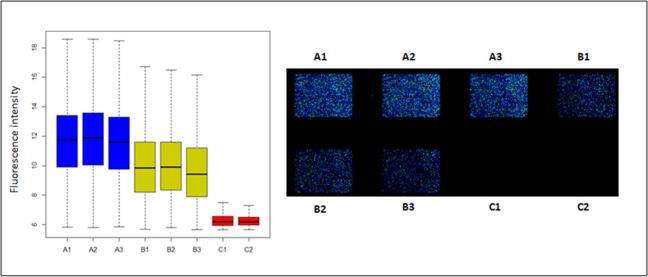
**Fluorescence signal of hybridization conditions on the microarray represented in boxplot (left) and the microarray slide (right).** Control group (A1, A2 and A3 in both cases) corresponds to the three experimental replicates of planktonic *Porphyromonas gingivalis* ATCC 33277. Test group (B1, B2 and B3 in both cases) represents the multispecies biofilm including *P*. *gingivalis*. And the negative control group (C1 and C2 in both cases) shows the multispecies-biofilm without *P*. *gingivalis*.

Fig 3 depicts the scanning glass slide of the microarray [Agilent *P*. *gingivalis* Oligo Microarrays 8x15K (074976)] and the fluorescence intensity measured after the hybridization of the three experimental replicates per group. Positive fluorescence was observed for *P*. *gingivalis* in planktonic growth (control group) (Fig 3 A1-A3) as well as within the multispecies biofilm (test group) (Fig 3 B1-B3). In contrast, there was no fluorescence in the negative control group, when *P*. *gingivalis* was not part of the multispecies biofilm (Fig 3 C1-C2), which confirmed the high specificity of the microarray without any sign of cross-hybridization.

**Fig 4:**
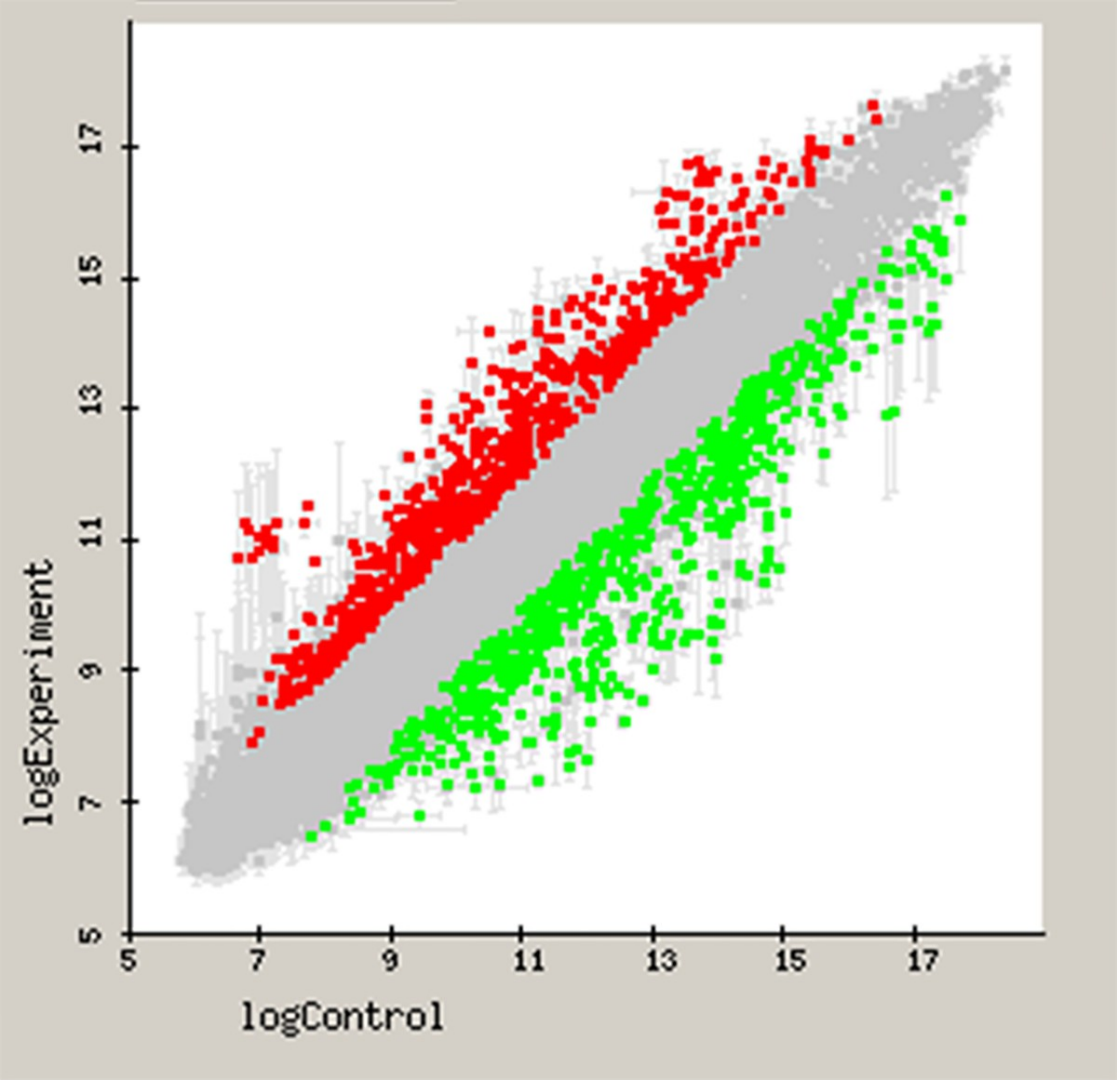
Microarray-based comparative transcriptome demonstrating the gene expression (represented in log_10_) for *Porphyromonas gingivalis* ATCC 33277 when growing in a multispecies biofilm compared to planktonic growth. X-axis depicts the fold difference in gene expression of *P*. *gingivalis* in planktonic growth, and the Y-axis the gene expression of *P*. *gingivalis* inside a multispecies-biofilm. Up-regulated genes (over-expressed in multispecies-biofilm) were represented as red color and down-regulated genes were colored in green.

Fig 4 depicts the *P*. *gingivalis* gene differential expression generated by the microarray (expressed as log_10_ of fluorescence), when growing in planktonic (X-axis, control) or within a multispecies biofilm (Y-axis, test). Using a linear model (LIMMA) with a filtering criterion of two-fold change (up or down) and significance p-value <0.05, a total of 365 out of 1,909 genes (19.1%) were found to have a differential expression when the two growing conditions were compared (S1 Table).

**Fig 5:**
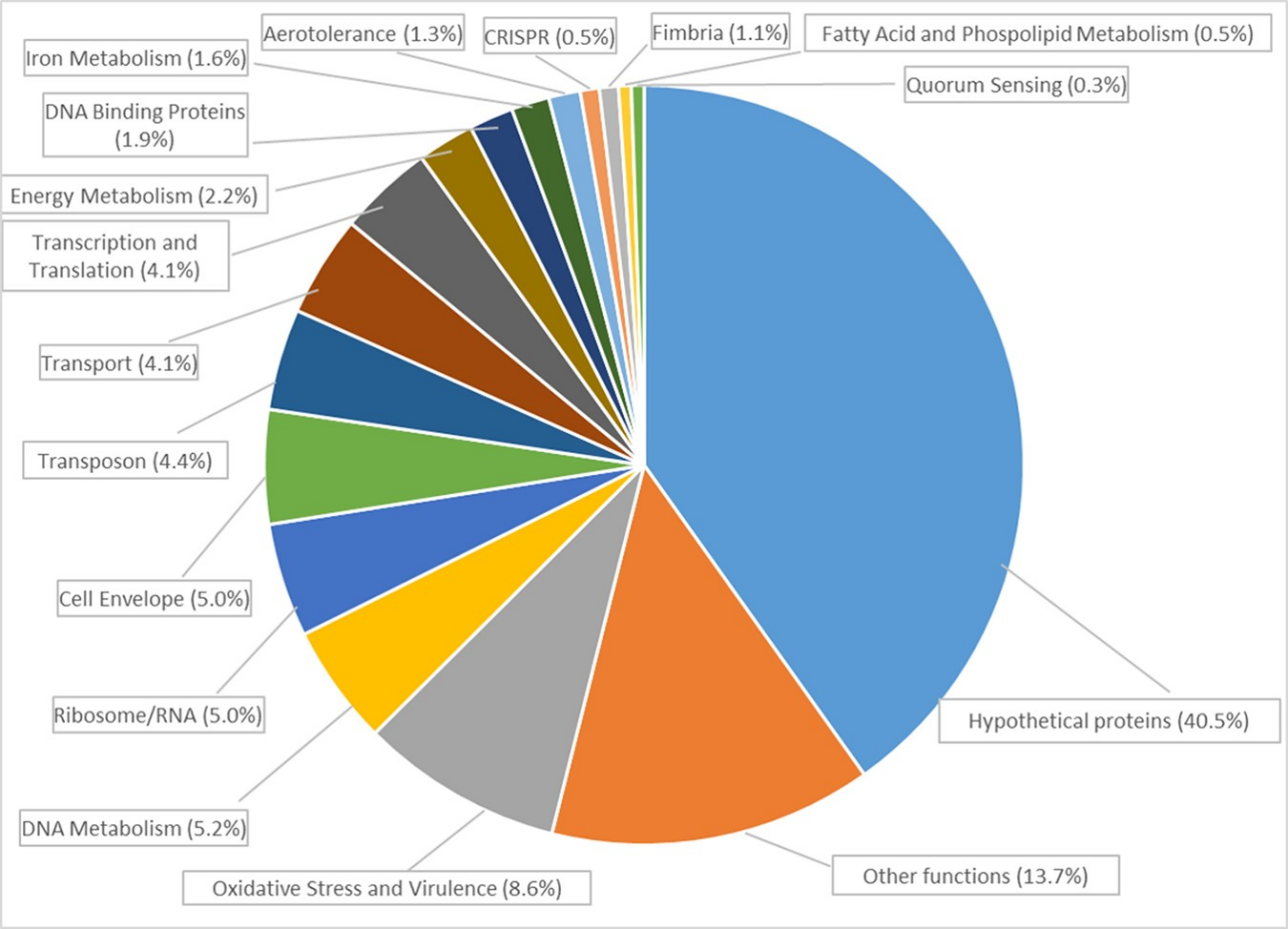
Distribution in functional categories of the differentially regulated genes of *Porphyromonas gingivalis* ATCC 33277 in planktonic cells compared to *P*. *gingivalis* within a multispecies-biofilm.

The *P*. *gingivalis* differentially expressed genes could be categorized in functional groups as depicted in Fig 5.

The complete list of 365 genes can be found in S1 Table. Of them, when *P*. *gingivalis* grew within a multispecies biofilm (test group), 165 genes were identified as up-regulated. These genes were mainly related to:

- **oxidative stress protection and secretion of virulence factors**, such as *SodB* superoxide dismutase [+5.71 (SD = 2.33)], thioredoxin system PGN_0033 [+7.33 (SD = 1.03)] and PG_0275 [+3.54 (SD = 1.11)], thiol peroxidases PG_1729 [+5.04 (SD = 0.74)] and PGN_0388 [+4.75 (SD = 1.01)], heat shock proteins as PGN_0041 [+2.58 (SD = 0.25)] and *Clp* system PGN_1208 [+8.31 (SD = 1.75)] and PGN_0008 [+3.01 (SD = 0.24)], chaperones *GroES* PGN_1451 [+7.93 (SD = 0.94) ] and *GrpE* PGN_1715 [+2.95 (SD = 0.50)];
- **cell–cell communication**, such as the gene encoding for quorum sensing S-adenosylmethionine synthase (PGN_1827) [+2.55 (SD = 0.26)];
- **iron metabolism**, as ferritin PGN_1058 [+6.11 (SD = 1.48)] and PGN_0604 [+7.28 (SD = 1.54)] and ferrodoxin PG_1813 [+2.25 (SD = 0.24)], *HmuY* [+3.29 (SD = 0.49)], PGN_0741 [+2.41 (SD = 0.38)];
- **ribosomes**, as *RpsA*, *RpsP*, *RpsT*, *RpmF*, *RpmH*, *RpmL*, *RplQ*, *RplT*, PG_0627 [+2.47 (SD = 0.05)] and PGN_0668 [+2.42 (SD = 0.17)];
- Other important functional genes, like the **transposon genes** that allow for adaptation to life in communities (*TraA-Q*) and CRISPR (*Cas*2-2 PGN_1959 [+4.80 (SD = 0.50)]).

When *P*. *gingivalis* grew within a multispecies biofilm (test group), 200 genes were down-regulated (S1 Table). These genes were mainly related to:

- **cell envelope**, as membrane proteins PG_0922 [-7.29 (SD = 2.61)], PG_1180 [-2.48 (SD = 0.59)], PG_2224 [-5.33 (SD = 0.13)], PGN_1020 [-4.36 (SD = 0.83)];
- **lipoproteins**, as PG_0180 [-3.44 (SD = 0.56)], PG_0399 [-2.34 (SD = 0.29)], PG_2133 [-14.42 (SD = 3.64)], PG_0924 [-5.03 (SD = 0.80)];
- **transport**, as ABC transporters PG_0912 [-2.36 (SD = 0.05)], PGN_1898 *MgtE* [-2.24 (SD = 0.27)], PGN_1876 [-2.41 (SD = 0.18)], PG_1010 [-2.57 (SD = 0.23)], PGN_1343 [-2.81 (SD = 0.71)], PGN_1734 [-3.09 (SD = 0.43)];
- **aerotolerance**, as (*BatA-E*), PGN_0529 [-5.86 (SD = 1.09)], PGN_0528 [-5.15 (SD = 1.70)], PGN_0527 [-5.25 (SD = 0.65)], PGN_0526 [-4.74 (SD = 1.14)], PGN_0525 [-8.28 (SD = 0.63)]
- **fimbria**, as *FimA* [-11.03 (SD = 1.71)], *FimC* [-17.73 (SD = 2.28)], *FimD* [-9.65 (SD = 1.85)].

Of the 365 differentially regulated *P*. *gingivalis* genes, 40.5% encoded for unknown function or hypothetical proteins (S1 Table).

**Fig 6:**
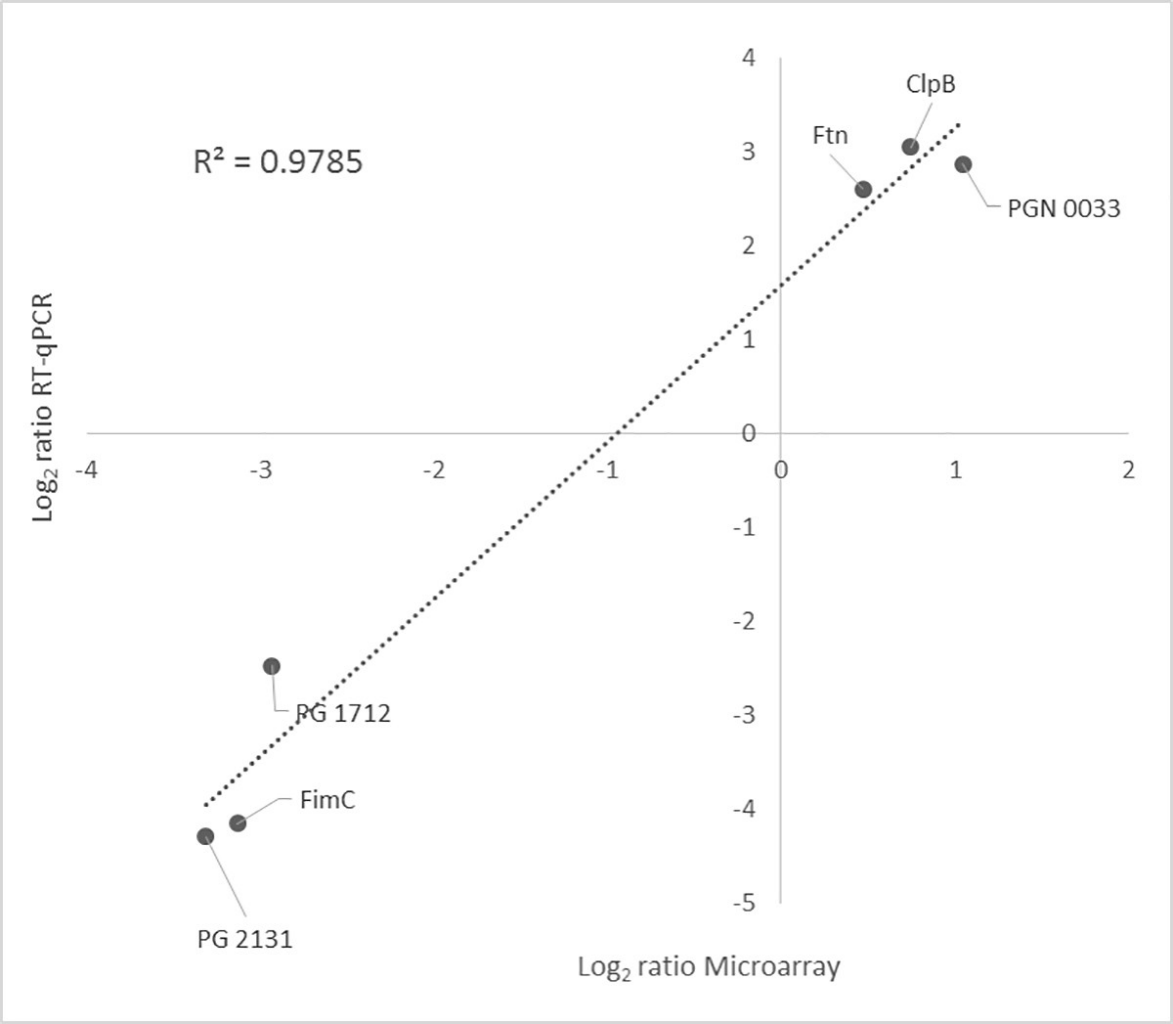
Correlation between Microarray and Reverse Transcription-quantitative Polymerase Chain Reaction (RT-qPCR) gene expression ratios when comparing *Porphyromonas gingivalis* ATCC 33277 growing within a multispecies-biofilm *versus* planktonic growth. RT-qPCR log_2_ values were plotted against the microarray data log_2_ values (R^2^ = 0.9785).

RT-qPCR confirmed the microarray results in three of the highly up-regulated genes and three of the highly down-regulated. Fig 6 depicts the log_2_ expression ratios for each technique demonstrating a high correlation between both (R^2^ = 0.9785).

## Discussion

The results from this *in vitro* investigation have revealed significant transcriptional changes when *P*. *gingivalis* grew within a multispecies biofilm, compared to planktonic growth, with 19.1% of *P*. *gingivalis* genes differentially expressed (165 genes were up-regulated and 200 down-regulated). The complete list of 365 genes can be found as Supporting Information file (S1 Table). In a previous report [14], we showed that gene expression of *P*. *gingivalis* changed from free floating to sessile, but the present study demonstrates that when biofilm conditions become more complex, as it is a multispecies microbial community, gene expression changes significantly enhanced inside the functional categories. In agreement with previous reports [9, 36], this polymicrobial synergy may increase the ability of *P*. *gingivalis* to colonize/predominate in the presence of other bacteria and thus increase its pathogenicity [22].

When compared to planktonic growth, *P*. *gingivalis* within a multispecies biofilm had significant differential expression in relevant groups of genes related to different functions and pathogenic pathways:

### Oxidative stress and virulence

The gene encoding the enzyme superoxide dismutase (*SodB)* was significantly up-regulated. This enzyme is responsible for transforming Reactive Oxygen Species (ROS) into peroxides, produced even in anaerobic conditions as a result of its own metabolism [37–39]. After that, peroxides are then generally disposed of by the enzyme catalase [40]. However, *P*. *gingivalis* cannot synthetize catalase and usually eliminates peroxides through the enzyme alkyl hydroperoxide reductase (*Ahp C–F*) [41]. Even though *AhpF* (PGN_0661) was down-regulated in the present study, the thioredoxin system (PGN_0033, PG_0275) and thiol peroxidase-encoding genes (PG_1729, PGN_0388), which are other pathways of peroxide removal [42, 43], were up-regulated. This finding has also been previously reported [44].

It was also significant with the up-regulation of genes involved in protein regulatory systems, such as heat shock proteins (Hsp), Clp proteolytic system *(ClpB*, *ClpC)* and the chaperones *(GroES*, *GrpE)*, which are usually expressed during stresses situations [45]. Clp proteases and chaperones are secreted by many pathogenic bacteria [45, 46] and they are involved in processes of colonization and adaptation to stress conditions. In fact, different bacterial species have shown attenuated virulence, reduced adhesion and biofilm formation when Clp proteins were mutated [47–53].

The complex (GroEL/GroES) is also relevant in terms of virulence, since these proteins can mediate in adhesion and attachment to the host cells [54–56]. Moreover, diverse inhibitors of GroEL/GroES are currently being tested as broad-spectrum antimicrobial agents [57, 58]. Hosogi and Duncan (2005) showed that GroEL and other Hsp mediated the entry of *P*. *gingivalis* into host epithelial cells [59]. *Porphyromonas gingivalis* GroEL was inhibited by immunization, which significantly reduced levels of alveolar bone loss in experimental animal model [60]. Llama-Palacios *et al*., in a proteomic study of *A*. *actinomycetemcomitans* biofilms, also reported increased expression in GroEL, and sera from patients with periodontitis were shown to be immunoreactive against GroEL [61].

Another potential pathogenic mechanism of GroEL proteins is their structural similarity with human Hsp. When overexpressed, GroEL may cause cross-reaction against human Hsp, causing autoantibodies and leading to chronic inflammation [53, 62].

Another important group of genes also found overexpressed in our study were those encoding for proteases, (PG_1060, PGN_1914 and PGN_0952) and peptidases (PG_0088, PGN_2035, PGN_1103, PGN_0788 and PG_1313). These proteases have been related to virulence, since they are able to degrade antibodies and, thus, to evade host tissue defenses and also to be involved in periodontal tissue destruction [63].

When the transcriptomic profile of *P*. *gingivalis* was studied when growing in a monospecies biofilms [14], only PGN_1914 was up-regulated, which may indicate that the up-regulation of the rest of the described proteases is mainly driven by the presence of other species within the biofilm.

### Cell envelope

The present transcriptomic study has revealed differential expression of those genes involved in cell envelope (PG_0679, PG_0922, PG_1039, PG_1180, PG_2224 and PGN_1020). PG_0679 was significantly up-regulated, what is in agreement with previous reports [13]. This gene is associated with antimicrobial resistance in multispecies biofilms, since efflux transporters pump out antimicrobial molecules [64].

However, *P*. *gingivalis* within a multispecies biofilm showed most of these genes down-regulated, which contrasts with previous reports studying *P*. *gingivalis* in monospecies biofilms [11, 14]. For example, the putative membrane protein gene PG_1180 and the putative epithelial cell attachment gene PG_2224, were significantly down-regulated.

Although the function is putative or not so concise, in general, the down-regulation of many genes involved in cell envelope biogenesis, taken together, with the down-regulation of metabolism genes involved in energy production or DNA replication, suggest a down-turn in cell replication and a reduced growth rate in biofilm. It has been previously attributed to restricted penetration of nutrients and helps explain the relative resistance of biofilms to antibiotics targeting growth [11]

Genes related with cell envelope lipoproteins, such as PG_0180, PG_0399, PG_1767, PG_1828, PG_2105, PG_2133 and PG_0924, were also differentially expressed. Among them, PG_1828 has been described as a strong cell activator, being major virulence factor for enhancing inflammatory responses [65]. In fact, inhibition of its activity by a deficient mutant suggests a direct link of PG_1828 in the pathogenesis of periodontitis [65].

### Quorum sensing

The gene PGN_1827 (*MetK)* demonstrated a significantly higher expression in *P*. *gingivalis* when growing in a multispecies biofilm. This gene encodes a protein related to the radical S-adenosyl-l-methionine (SAM) superfamily, the universal signal for quorum sensing (QS). These proteins are responsible of the biosynthetic pathways leading to autoinducer-2 (AI-2) production, which are key in cell-cell communication what affects different bacterial functions related to virulence, such as motility, nutrition, phenotype expressions and modulation, stability and composition of the biofilms [9, 66–69].

### Iron metabolism

The present study has shown a significant up-regulation of genes as PGN_0741, a TonB-dependent outer membrane receptor important for iron transportation [70] and *HmuY*. *Hmu* family proteins are important for hemin acquisition, which is key for *P*. *gingivalis*. This bacterium has an absolute growth requirement for hemin, which provides them with iron and protoporphyrin IX that cannot be synthesize by itself [41, 71]. *Hmu* family genes has also been implicated as a virulence factor in promoting mononuclear cell-mediated inflammatory responses [72, 73]. The up-regulation of these genes, and in this case *HmuY*,can influence the ability of *P*. *gingivalis* to promote biofilm formation, as was seen in our previous study, in which the gene *HmuR* was up-regulated when growing in planktonic form in presence of a growing monospecies biofilm [23].

There was also a significant up-regulation of the genes coding for ferritin and ferrodoxin (*Ftn*, PGN_0604, PG_1813). Ferritin has been shown to be a requirement for *P*. *gingivalis* to grow under iron-depleted conditions and peroxide stress [42, 74].

### Transposons and CRISPR

*P*. *gingivalis* growing within a multispecies biofilm demonstrated up-regulation of several transposons genes (*TraA*, *TraF*, *TraG*, *TraI*, *TraJ*, *TraK*, *TraM*, *TraN*, *TraO*, *TraP*, *TraQ*, PGN_0058, PGN_0056, PG_1061, PGN_0954 and PGN_1912), suggesting that *P*. *gingivalis*, when growing among competing species, develops horizontal DNA transfer, which may facilitate its adaptation to different micro-environments. This up-regulation has also been reported in other studies [75–77]. Again, these genes were also up-regulated when planktonic *P*. *gingivalis* was in the presence of a growing biofilm, what may indicate the importance of DNA transfer to allow for survival in different environments and to adapt to an evolving biofilm [23].

Similarly, the genes of the CRISPR system: *Cas*2-2 and PGN_1959 were up-regulated in the test group. These genes encode for a CRISPR protein related to CAS2 family, which play broad roles in controlling bacterial pathogenesis, gene regulation and physiology [78], and protect its genome against other surrounding microorganisms and mobile foreign genetic elements, in particular plasmids and transposons [79–81].

Among other functional groups, genes related to ribosome, as *RpsA*, *RpsP*, *RpsT*, *RpmF*, *RpmH*, *RpmL*, *RplQ*, *RplT*, PG_0627 and PGN_0668, were up-regulated in the test group, what presumably indicates increased translation and higher protein synthesis. The fimbria genes *FimA*, *FimC*, *FimD* were down-regulated in *P*. *gingivalis* within the multispecies biofilm. This fact has also been reported when *P*. *gingivalis* grew in monospecies biofilm [14], or when planktonic *P*. *gingivalis* grew in the presence of a monospecies biofilm [23]. Other authors have reported that FimA and Mfa1 were not required for the development of pathogenicity in biofilms [82, 83]. A large proportion of the genes in (S1 Table) of the Supporting Information, demonstrating differential expression when comparing both groups, were however related to proteins of unknown function (40.5%), what indicates the need to further research to understand the functionality of these genes.

This study has clear limitations, such as its *in vitro* nature, the limited number of bacterial species used to develop the biofilms or the lack of influence of the patient's immune response as well as its physiological condition, which would also influence the gene expression of *P*. *gingivalis*. Hence, further studies are needed to ascertain the pathogenic capability of *P*. *gingivalis* in the initiation and progression of periodontitis, as well as the transcriptomic changes that *P*. *gingivalis* could suffer accompanied by the other species when growing also in planktonic state.

## Conclusions

This study has shown that 19.1% of the *P*. *gingivalis* genome was differentially expressed when it grew within a multispecies biofilm, in comparison with monospecies planktonic growth. Within the biofilm, *P*. *gingivalis* has shown increased expression of virulence factors and antioxidant enzymes, especially Hsp proteins and several proteases. The identification and quantification of these known genes and other related to unknown proteins may provide new knowledge on the virulence and pathogenicity of this important periodontal pathogen, *P*. *gingivalis*.

## Supporting information

S1 Table Differentially expressed genes in *Porphyromonas gingivalis* ATCC 33277 in planktonic condition and within a multispecies-biofilm (cutoff ratio ≥ ±2; p-value <0.05), grouped by functional categories. Microarray data indicate the mean expression fold change and Standard Deviation (SD) of each gene.
(DOCX)

## References

[1] Paster BJ, Boches SK, Galvin JL, Ericson RE, Lau CN, Levanos VA, et al Bacterial diversity in human subgingival plaque. Journal of bacteriology. 2001;183(12):3770–83. Epub 2001/05/24. 10.1128/JB.183.12.3770-3783.2001 11371542 PMC95255

[2] Park JH, Lee JK. A periodontitis-associated multispecies model of an oral biofilm. Journal of periodontal & implant science. 2014;44(2):79–84. 10.5051/jpis.2014.44.2.79 . 24778902 PMC3999356

[3] Redanz S, Standar K, Podbielski A, Kreikemeyer B. A five-species transcriptome array for oral mixed-biofilm studies. PloS one. 2011;6(12):e27827 Epub 2011/12/24. 10.1371/journal.pone.0027827 22194794 PMC3237422

[4] Donlan RM. Biofilms: microbial life on surfaces. Emerging infectious diseases. 2002;8(9):881–90. Epub 2002/08/27. 10.3201/eid0809.020063 12194761 PMC2732559

[5] Socransky SS, Haffajee AD. Dental biofilms: difficult therapeutic targets. Periodontology 2000. 2002;28:12–55. Epub 2002/05/16. . 12013340 10.1034/j.1600-0757.2002.280102.x

[6] Limoli DH, Jones CJ, Wozniak DJ. Bacterial Extracellular Polysaccharides in Biofilm Formation and Function. Microbiology spectrum. 2015;3(3):10.1128/microbiolspec.MB-0011-2014. 10.1128/microbiolspec.MB-0011-2014 PMC4657554. 26185074 PMC4657554

[7] Marsh PD. Dental plaque as a biofilm and a microbial community—implications for health and disease. BMC oral health. 2006;6 Suppl 1:S14 Epub 2006/08/29. 10.1186/1472-6831-6-s1-s14 16934115 PMC2147593

[8] Sanz M, Beighton D, Curtis MA, Cury JA, Dige I, Dommisch H, et al Role of microbial biofilms in the maintenance of oral health and in the development of dental caries and periodontal diseases. Consensus report of group 1 of the Joint EFP/ORCA workshop on the boundaries between caries and periodontal disease. Journal of clinical periodontology. 2017;44 Suppl 18:S5–s11. Epub 2017/03/08. 10.1111/jcpe.12682 . 28266109

[9] Hajishengallis G, Lamont RJ. Beyond the red complex and into more complexity: the polymicrobial synergy and dysbiosis (PSD) model of periodontal disease etiology. Molecular oral microbiology. 2012;27(6):409–19. 10.1111/j.2041-1014.2012.00663.x PMC3653317. 23134607 PMC3653317

[10] Hajishengallis G, Lamont RJ. Breaking bad: manipulation of the host response by *Porphyromonas gingivalis*. European journal of immunology. 2014;44(2):328–38. Epub 2013/12/18. 10.1002/eji.201344202 24338806 PMC3925422

[11] Lo AW, Seers CA, Boyce JD, Dashper SG, Slakeski N, Lissel JP, et al Comparative transcriptomic analysis of *Porphyromonas gingivalis* biofilm and planktonic cells. BMC microbiology. 2009;9:18 Epub 2009/01/30. 10.1186/1471-2180-9-18 19175941 PMC2637884

[12] Yamamoto R, Noiri Y, Yamaguchi M, Asahi Y, Maezono H, Ebisu S. Time course of gene expression during *Porphyromonas gingivalis* strain ATCC 33277 biofilm formation. Applied and environmental microbiology. 2011;77(18):6733–6. Epub 2011/08/02. 10.1128/AEM.00746-11 21803908 PMC3187161

[13] Hovik H, Yu WH, Olsen I, Chen T. Comprehensive transcriptome analysis of the periodontopathogenic bacterium *Porphyromonas gingivalis* W83. Journal of bacteriology. 2012;194(1):100–14. Epub 2011/11/01. 10.1128/JB.06385-11 22037400 PMC3256594

[14] Romero-Lastra P, Sanchez MC, Ribeiro-Vidal H, Llama-Palacios A, Figuero E, Herrera D, Sanz M. Comparative gene expression analysis of *Porphyromonas gingivalis* ATCC 33277 in planktonic and biofilms states. PloS one. 2017;12(4):e0174669 Epub 2017/04/04. 10.1371/journal.pone.0174669 28369099 PMC5378342

[15] Liu D, Xu J, Wang Y, Chen Y, Shen X, Niu H, et al Comparative transcriptomic analysis of *Clostridium acetobutylicum* biofilm and planktonic cells. Journal of biotechnology. 2016;218:1–12. Epub 2015/12/02. 10.1016/j.jbiotec.2015.11.017 . 26621081

[16] Schembri MA, Kjaergaard K, Klemm P. Global gene expression in *Escherichia coli* biofilms. Molecular microbiology. 2003;48(1):253–67. Epub 2003/03/27. 10.1046/j.1365-2958.2003.03432.x . 12657059

[17] Beenken KE, Dunman PM, McAleese F, Macapagal D, Murphy E, Projan SJ, et al Global gene expression in *Staphylococcus aureus* biofilms. Journal of bacteriology. 2004;186(14):4665–84. Epub 2004/07/03. 10.1128/JB.186.14.4665-4684.2004 15231800 PMC438561

[18] Lamont RJ, Jenkinson HF. Life below the gum line: pathogenic mechanisms of *Porphyromonas gingivalis*. Microbiology and molecular biology reviews. 1998;62(4):1244–63. Epub 1998/12/05. 9841671 10.1128/mmbr.62.4.1244-1263.1998 PMC98945

[19] Marsh PD. Dental plaque as a microbial biofilm. Caries research. 2004;38(3):204–11. Epub 2004/05/22. 10.1159/000077756 . 15153690

[20] Socransky SS, Haffajee AD. The bacterial etiology of destructive periodontal disease: current concepts. Journal of periodontology. 1992;63(4 Suppl):322–31. Epub 1992/04/01. 10.1902/jop.1992.63.4s.322 . 1573546

[21] Guggenheim B, Gmur R, Galicia JC, Stathopoulou PG, Benakanakere MR, Meier A, et al *In vitro* modeling of host-parasite interactions: the 'subgingival' biofilm challenge of primary human epithelial cells. BMC microbiology. 2009;9:280 Epub 2010/01/02. 10.1186/1471-2180-9-280 20043840 PMC2818713

[22] Frias-Lopez J, Duran-Pinedo A. Effect of periodontal pathogens on the metatranscriptome of a healthy multispecies biofilm model. Journal of bacteriology. 2012;194(8):2082–95. Epub 2012/02/14. 10.1128/JB.06328-11 22328675 PMC3318478

[23] Sanchez MC, Romero-Lastra P, Ribeiro-Vidal H, Llama-Palacios A, Figuero E, Herrera D, Sanz M. Comparative gene expression analysis of planktonic *Porphyromonas gingivalis* ATCC 33277 in the presence of a growing biofilm versus planktonic cells. BMC microbiology. 2019;19(1):58 Epub 2019/03/15. 10.1186/s12866-019-1423-9 . 30866810 PMC6417203

[24] Díaz PI, Kolenbrander PE. Subgingival Biofilm Communities in Health and Disease. Revista Clínica de Periodoncia, Implantología y Rehabilitación Oral. 2009;2(3):187–92. 10.1016/S0718-5391(09)70033-3.

[25] Kolenbrander PE, Palmer RJ Jr., Periasamy S, Jakubovics NS. Oral multispecies biofilm development and the key role of cell-cell distance. Nature reviews Microbiology. 2010;8(7):471–80. Epub 2010/06/02. 10.1038/nrmicro2381 . 20514044

[26] Kuboniwa M, Lamont RJ. Subgingival biofilm formation. Periodontology 2000. 2010;52(1):38–52. Epub 2009/12/19. 10.1111/j.1600-0757.2009.00311.x 20017794 PMC3665295

[27] Kuboniwa M, Houser JR, Hendrickson EL, Wang Q, Alghamdi SA, Sakanaka A. Metabolic crosstalk regulates *Porphyromonas gingivalis* colonization and virulence during oral polymicrobial infection. Nature microbiology. 2017;2(11):1493–9. 10.1038/s41564-017-0021-6 . 28924191 PMC5678995

[28] Bao K, Belibasakis GN, Thurnheer T, Aduse-Opoku J, Curtis MA, Bostanci N. Role of *Porphyromonas gingivalis* gingipains in multi-species biofilm formation. BMC microbiology. 2014;14:258 Epub 2014/10/02. 10.1186/s12866-014-0258-7 25270662 PMC4189655

[29] Bakthavatchalu V, Meka A, Mans JJ, Sathishkumar S, Lopez MC, Bhattacharyya I, et al Polymicrobial periodontal pathogens transcriptomes in calvarial bone and soft tissue. Molecular oral microbiology. 2011;26(5):303–20. 10.1111/j.2041-1014.2011.00619.x PMC3170131. 21896157 PMC3170131

[30] Brogden KA, Guthmiller JM, Taylor CE. Human polymicrobial infections. Lancet (London, England). 2005;365(9455):253–5. Epub 2005/01/18. 10.1016/s0140-6736(05)17745-9 . 15652608 PMC7119324

[31] Sanchez MC, Llama-Palacios A, Blanc V, Leon R, Herrera D, Sanz M. Structure, viability and bacterial kinetics of an *in vitro* biofilm model using six bacteria from the subgingival microbiota. Journal of periodontal research. 2011;46(2):252–60. Epub 2011/01/26. 10.1111/j.1600-0765.2010.01341.x . 21261622

[32] Smyth GK, Speed T. Normalization of cDNA microarray data. Methods (San Diego, Calif). 2003;31(4):265–73. Epub 2003/11/05. . 14597310 10.1016/s1046-2023(03)00155-5

[33] Smyth GK. Linear models and empirical bayes methods for assessing differential expression in microarray experiments. Statistical applications in genetics and molecular biology. 2004;3:Article3. Epub 2006/05/02. 10.2202/1544-6115.1027 . 16646809

[34] Ihaka R, Gentleman R. R: A language for data analysis and graphics. Journal of Computational and Graphical Statistics. 1996;5(3):299–314.

[35] Schmittgen TD, Livak KJ. Analyzing real-time PCR data by the comparative C(T) method. Nature protocols. 2008;3(6):1101–8. Epub 2008/06/13. . 18546601 10.1038/nprot.2008.73

[36] Comolli LR. Intra- and inter-species interactions in microbial communities. Frontiers in Microbiology. 2014;5:629 10.3389/fmicb.2014.00629 PMC4241841. 25505455 PMC4241841

[37] Nakayama K. Rapid viability loss on exposure to air in a superoxide dismutase-deficient mutant of *Porphyromonas gingivalis*. Journal of bacteriology. 1994;176(7):1939–43. Epub 1994/04/01. 10.1128/jb.176.7.1939-1943.1994 8144460 PMC205297

[38] Imlay JA. Cellular defenses against superoxide and hydrogen peroxide. Annual review of biochemistry. 2008;77:755–76. Epub 2008/01/05. 10.1146/annurev.biochem.77.061606.161055 18173371 PMC3057177

[39] Henry LG, McKenzie RME, Robles A, Fletcher HM. Oxidative stress resistance in *Porphyromonas gingivalis*. Future microbiology. 2012;7(4):497–512. 10.2217/fmb.12.17 PMC3397238. 22439726 PMC3397238

[40] Wang Y, Branicky R, Noe A, Hekimi S. Superoxide dismutases: Dual roles in controlling ROS damage and regulating ROS signaling. 2018;217(6):1915–28. 10.1083/jcb.201708007 . 29669742 PMC5987716

[41] Henry LG, McKenzie RM, Robles A, Fletcher HM. Oxidative stress resistance in *Porphyromonas gingivalis*. Future microbiology. 2012;7(4):497–512. Epub 2012/03/24. 10.2217/fmb.12.17 22439726 PMC3397238

[42] Lewis JP, Iyer D, Anaya-Bergman C. Adaptation of *Porphyromonas gingivalis* to microaerophilic conditions involves increased consumption of formate and reduced utilization of lactate. Microbiology (Reading, England). 2009;155(Pt 11):3758–74. Epub 2009/08/18. 10.1099/mic.0.027953-0 19684063 PMC2888126

[43] Wan XY, Zhou Y, Yan ZY, Wang HL, Hou YD, Jin DY. Scavengase p20: a novel family of bacterial antioxidant enzymes. FEBS letters. 1997;407(1):32–6. Epub 1997/04/21. 10.1016/s0014-5793(97)00302-5 . 9141476

[44] Moon JH, Lee JH, Lee JY. Microarray analysis of the transcriptional responses of *Porphyromonas gingivalis* to polyphosphate. BMC microbiology. 2014;14:218 Epub 2014/08/26. 10.1186/s12866-014-0218-2 25148905 PMC4236598

[45] Capestany CA, Tribble GD, Maeda K, Demuth DR, Lamont RJ. Role of the Clp system in stress tolerance, biofilm formation, and intracellular invasion in *Porphyromonas gingivalis*. Journal of bacteriology. 2008;190(4):1436–46. Epub 2007/12/11. 10.1128/JB.01632-07 18065546 PMC2238200

[46] Kajfasz JK, Martinez AR, Rivera-Ramos I, Abranches J, Koo H, Quivey RG Jr., et al Role of Clp proteins in expression of virulence properties of Streptococcus mutans. Journal of bacteriology. 2009;191(7):2060–8. Epub 2009/02/03. 10.1128/JB.01609-08 19181818 PMC2655509

[47] Butler SM, Festa RA, Pearce MJ, Darwin KH. Self-compartmentalized bacterial proteases and pathogenesis. Molecular microbiology. 2006;60(3):553–62. Epub 2006/04/25. 10.1111/j.1365-2958.2006.05128.x . 16629660

[48] Henderson B, Allan E, Coates ARM. Stress Wars: the Direct Role of Host and Bacterial Molecular Chaperones in Bacterial Infection. Infection and Immunity. 2006;74(7):3693–706. 10.1128/IAI.01882-05 PMC1489680. 16790742 PMC1489680

[49] Frees D, Chastanet A, Qazi S, Sorensen K, Hill P, Msadek T, et al Clp ATPases are required for stress tolerance, intracellular replication and biofilm formation in *Staphylococcus aureus*. Molecular microbiology. 2004;54(5):1445–62. Epub 2004/11/24. 10.1111/j.1365-2958.2004.04368.x . 15554981

[50] Lemos JA, Burne RA. Regulation and Physiological Significance of ClpC and ClpP in *Streptococcus mutans*. Journal of bacteriology. 2002;184(22):6357–66. Epub 2002/10/26. 10.1128/JB.184.22.6357-6366.2002 12399506 PMC151938

[51] Rouquette C, de Chastellier C, Nair S, Berche P. The ClpC ATPase of *Listeria monocytogenes* is a general stress protein required for virulence and promoting early bacterial escape from the phagosome of macrophages. Molecular microbiology. 1998;27(6):1235–45. Epub 1998/05/07. 10.1046/j.1365-2958.1998.00775.x . 9570408

[52] Yuan L, Rodrigues PH, Belanger M, Dunn W Jr., Progulske-Fox A. The *Porphyromonas gingivalis* clpB gene is involved in cellular invasion *in vitro* and virulence in vivo. FEMS immunology and medical microbiology. 2007;51(2):388–98. Epub 2007/09/15. 10.1111/j.1574-695X.2007.00326.x . 17854400

[53] Yuan L, Hillman JD, Progulske-Fox A. Microarray analysis of quorum-sensing-regulated genes in *Porphyromonas gingivalis*. Infection and immunity. 2005;73(7):4146–54. Epub 2005/06/24. 10.1128/IAI.73.7.4146-4154.2005 15972504 PMC1168601

[54] Mohammed MMA, Pettersen VK, Nerland AH, Wiker HG, Bakken V. Quantitative proteomic analysis of extracellular matrix extracted from mono- and dual-species biofilms of *Fusobacterium nucleatum* and *Porphyromonas gingivalis*. Anaerobe. 2017;44:133–42. Epub 2017/03/13. 10.1016/j.anaerobe.2017.03.002 . 28285095

[55] Lewthwaite J, Skinner A, Henderson B. Are molecular chaperones microbial virulence factors? Trends in microbiology. 1998;6(11):426–8. Epub 1998/12/10. . 9846353 10.1016/s0966-842x(98)01362-6

[56] Hennequin C, Porcheray F, Waligora-Dupriet A, Collignon A, Barc M, Bourlioux P, et al GroEL (Hsp60) of *Clostridium difficile* is involved in cell adherence. Microbiology (Reading, England). 2001;147(Pt 1):87–96. Epub 2001/02/13. 10.1099/00221287-147-1-87 . 11160803

[57] Johnson SM, Sharif O, Mak PA, Wang HT, Engels IH, Brinker A, et al A biochemical screen for GroEL/GroES inhibitors. Bioorganic & medicinal chemistry letters. 2014;24(3):786–9. Epub 2014/01/15. 10.1016/j.bmcl.2013.12.100 . 24418775

[58] Skar CK, Kruger PG, Bakken V. Characterisation and subcellular localisation of the GroEL-like and DnaK-like proteins isolated from *Fusobacterium nucleatum* ATCC 10953. Anaerobe. 2003;9(6):305–12. Epub 2006/08/05. 10.1016/j.anaerobe.2003.08.004 . 16887717

[59] Hosogi Y, Duncan MJ. Gene expression in *Porphyromonas gingivalis* after contact with human epithelial cells. Infection and immunity. 2005;73(4):2327–35. Epub 2005/03/24. 10.1128/IAI.73.4.2327-2335.2005 15784578 PMC1087432

[60] Lee JY, Yi NN, Kim US, Choi JS, Kim SJ, Choi JI. *Porphyromonas gingivalis* heat shock protein vaccine reduces the alveolar bone loss induced by multiple periodontopathogenic bacteria. Journal of periodontal research. 2006;41(1):10–4. Epub 2006/01/18. 10.1111/j.1600-0765.2005.00832.x . 16409250

[61] Llama-Palacios A, Potupa O, Sanchez MC, Figuero E, Herrera D, Sanz M. *Aggregatibacter actinomycetemcomitans* Growth in Biofilm versus Planktonic State: Differential Expression of Proteins. Journal of proteome research. 2017;16(9):3158–67. Epub 2017/07/15. 10.1021/acs.jproteome.7b00127 . 28707473

[62] Lopatin DE, Shelburne CE, Van Poperin N, Kowalski CJ, Bagramian RA. Humoral immunity to stress proteins and periodontal disease. Journal of periodontology. 1999;70(10):1185–93. Epub 1999/10/26. 10.1902/jop.1999.70.10.1185 . 10534073

[63] Kadowaki T, Nakayama K, Okamoto K, Abe N, Baba A, Shi Y, et al *Porphyromonas gingivalis* proteinases as virulence determinants in progression of periodontal diseases. Journal of biochemistry. 2000;128(2):153–9. Epub 2000/08/02. 10.1093/oxfordjournals.jbchem.a022735 . 10920248

[64] Rahman T, Yarnall B, Doyle DA. Efflux drug transporters at the forefront of antimicrobial resistance. European biophysics journal: EBJ. 2017;46(7):647–53. Epub 07/14. 10.1007/s00249-017-1238-2 . 28710521 PMC5599465

[65] Asai Y, Hashimoto M, Fletcher HM, Miyake K, Akira S, Ogawa T. Lipopolysaccharide preparation extracted from *Porphyromonas gingivalis* lipoprotein-deficient mutant shows a marked decrease in toll-like receptor 2-mediated signaling. Infection and immunity. 2005;73(4):2157–63. 10.1128/IAI.73.4.2157-2163.2005 . 15784558 PMC1087447

[66] Kesavalu L, Sathishkumar S, Bakthavatchalu V, Matthews C, Dawson D, Steffen M, et al Rat model of polymicrobial infection, immunity, and alveolar bone resorption in periodontal disease. Infection and immunity. 2007;75(4):1704–12. Epub 2007/01/11. 10.1128/IAI.00733-06 17210663 PMC1865722

[67] Orth RK, O'Brien-Simpson NM, Dashper SG, Reynolds EC. Synergistic virulence of *Porphyromonas gingivalis* and *Treponema denticola* in a murine periodontitis model. Molecular oral microbiology. 2011;26(4):229–40. Epub 2011/07/07. 10.1111/j.2041-1014.2011.00612.x . 21729244

[68] Miller MB, Bassler BL. Quorum sensing in bacteria. Annual review of microbiology. 2001;55:165–99. Epub 2001/09/07. 10.1146/annurev.micro.55.1.165 . 11544353

[69] Parveen N, Cornell KA. Methylthioadenosine/S-adenosylhomocysteine nucleosidase, a critical enzyme for bacterial metabolism. Molecular microbiology. 2011;79(1):7–20. Epub 11/18. 10.1111/j.1365-2958.2010.07455.x . 21166890 PMC3057356

[70] Lewis JP, Plata K, Yu F, Rosato A, Anaya C. Transcriptional organization, regulation and role of the *Porphyromonas gingivalis* W83 hmu haemin-uptake locus. Microbiology (Reading, England). 2006;152(Pt 11):3367–82. Epub 2006/11/01. 10.1099/mic.0.29011-0 . 17074906

[71] Lewis JP. Metal uptake in host-pathogen interactions: role of iron in *Porphyromonas gingivalis* interactions with host organisms. Periodontology 2000. 2010;52(1):94–116. Epub 2009/12/19. 10.1111/j.1600-0757.2009.00329.x 20017798 PMC2825758

[72] Trindade SC, Olczak T, Gomes-Filho IS, de Moura-Costa LF, Vale VC, Galdino-Neto M, et al *Porphyromonas gingivalis* HmuY-induced production of interleukin-6 and IL-6 polymorphism in chronic periodontitis. Journal of periodontology. 2013;84(5):650–5. Epub 2012/07/10. 10.1902/jop.2012.120230 . 22769440

[73] Carvalho-Filho PC, Gomes-Filho IS, Meyer R, Olczak T, Xavier MT, Trindade SC. Role of *Porphyromonas gingivalis* HmuY in Immunopathogenesis of Chronic Periodontitis. Mediators of inflammation. 2016;2016:7465852 Epub 2016/07/13. 10.1155/2016/7465852 27403039 PMC4925967

[74] Ueshima J, Shoji M, Ratnayake DB, Abe K, Yoshida S, Yamamoto K, et al Purification, gene cloning, gene expression, and mutants of Dps from the obligate anaerobe *Porphyromonas gingivalis*. Infection and immunity. 2003;71(3):1170–8. Epub 2003/02/22. 10.1128/IAI.71.3.1170-1178.2003 12595429 PMC148816

[75] Tribble GD, Lamont GJ, Progulske-Fox A, Lamont RJ. Conjugal Transfer of Chromosomal DNA Contributes to Genetic Variation in the Oral Pathogen *Porphyromonas gingivalis*. Journal of bacteriology. 2007;189(17):6382–8. 10.1128/JB.00460-07 PMC1951918. 17573478 PMC1951918

[76] Hendrickson EL, Beck DA, Miller DP, Wang Q, Whiteley M, Lamont RJ, et al Insights into Dynamic Polymicrobial Synergy Revealed by Time-Coursed RNA-Seq. Front Microbiol. 2017;8:261 Epub 2017/03/16. 10.3389/fmicb.2017.00261 28293219 PMC5329018

[77] Mitchell HL, Dashper SG, Catmull DV, Paolini RA, Cleal SM, Slakeski N, et al *Treponema denticola* biofilm-induced expression of a bacteriophage, toxin-antitoxin systems and transposases. Microbiology (Reading, England). 2010;156(Pt 3):774–88. Epub 2009/12/17. 10.1099/mic.0.033654-0 . 20007650

[78] Sampson TR, Weiss DS. CRISPR-Cas systems: new players in gene regulation and bacterial physiology. Frontiers in cellular and infection microbiology. 2014;4:37 Epub 2014/04/29. 10.3389/fcimb.2014.00037 24772391 PMC3983513

[79] Barrangou R, Fremaux C, Deveau H, Richards M, Boyaval P, Moineau S, et al CRISPR provides acquired resistance against viruses in prokaryotes. Science (New York, NY). 2007;315(5819):1709–12. Epub 2007/03/24. 10.1126/science.1138140 . 17379808

[80] Marraffini LA, Sontheimer EJ. CRISPR interference limits horizontal gene transfer in *Staphylococci* by targeting DNA. Science (New York, NY). 2008;322(5909):1843–5. Epub 2008/12/20. 10.1126/science.1165771 19095942 PMC2695655

[81] Garneau JE, Dupuis ME, Villion M, Romero DA, Barrangou R, Boyaval P, et al The CRISPR/Cas bacterial immune system cleaves bacteriophage and plasmid DNA. Nature. 2010;468(7320):67–71. Epub 2010/11/05. 10.1038/nature09523 . 21048762

[82] Krogfelt KA, Klemm P. Investigation of minor components of *Escherichia coli* type 1 fimbriae: protein chemical and immunological aspects. Microbial pathogenesis. 1988;4(3):231–8. Epub 1988/03/01. . 2904111 10.1016/0882-4010(88)90073-3

[83] Nagano K, Hasegawa Y, Abiko Y, Yoshida Y, Murakami Y, Yoshimura F. *Porphyromonas gingivalis* FimA fimbriae: fimbrial assembly by fimA alone in the fim gene cluster and differential antigenicity among fimA genotypes. PloS one. 2012;7(9):e43722 Epub 2012/09/13. 10.1371/journal.pone.0043722 22970139 PMC3436787

