# Supplementary material for: Gene expression of Porphyromonas gingivalis ATCC 33277 when growing in an in vitro multispecies biofilm
Source: PLoS One. 2019 Aug 22;14(8):e0221234. doi: 10.1371/journal.pone.0221234 (PMC6706054; doi:10.1371/journal.pone.0221234)
Supplement: S1 Table — Microarray data indicate the mean expression fold change and Standard Deviation (SD) of each gene. (DOCX) [file pone.0221234.s001.docx]

**SUPPORTING INFORMATION**

**S1 Table. Differentially expressed genes in *Porphyromonas gingivalis* ATCC 33277 in planktonic condition and within a multispecies-biofilm (cutoff ratio ≥ ±2; p-value <0.05), grouped by functional categories. Microarray data indicate the mean expression fold change and Standard Deviation (SD) of each gene.**

| **OPEN READING FRAME** | **LOCUS NAME** | **PUTATIVE IDENTIFICATION** | **AVERAGE RELATIVE FOLD CHANGE (SD)** | | |
| --- | --- | --- | --- | --- | --- |
| **GENES RELATED TO OXIDATIVE STRESS AND VIRULENCE** | | | | | |
| **PGN_0564** | *SodB* | superoxide dismutase Fe-Mn | | +5.71 | (2.33) |
| **PGN_1055** | *VimE* | virulence modulating gene E | | -2.50 | (0.64) |
| **PGN_0033** | *Trx* | Thioredoxin | | +7.33 | (1.02) |
| **PG_0275** |  | thioredoxin family protein | | +3.54 | (1.11) |
| **PG_1729** |  | thiol peroxidase | | +5.04 | (0.74) |
| **PGN_0388** |  | putative thiol peroxidase | | +4.75 | (1.01) |
| **PGN_0661** |  | alkyl hydroperoxide reductase F subunit | | -3.89 | (0.55) |
| **PGN_1286** |  | probable lysozyme | | +3.02 | (0.37) |
| **PGN_0055** |  | probable lysozyme | | +2.45 | (0.23) |
| **PGN_0290** |  | immunoreactive 32 kDa antigen | | -3.55 | (0.02) |
| **PG_0181** |  | immunoreactive 32 kDa antigen PG49 | | -3.46 | (0.52) |
| **PGN_0152** |  | immunoreactive 61 kDa antigen | | -8.51 | (3.46) |
| **PG_2102** |  | immunoreactive 61 kDa antigen PG91 | | -6.44 | (1.06) |
| **PG_0234** |  | immunoreactive 23 kDa antigen PG66 | | +2.38 | (0.29) |
| **PGN_0482** |  | probable immunoreactive 23 kDa antigen | | -3.07 | (0.85) |
| **PGN_0041** | *HtpG* | heat shock protein HtpG | | +2.58 | (0.25) |
| **PGN_1208** | *ClpB* | ClpB protein | | +8.31 | (1.75) |
| **PGN_0008** | *ClpC* | ATP-dependent Clp protease ATP-binding subunit ClpC | | +3.01 | (0.24) |
| **PGN_1550** | *ClpX* | ATP-dependent Clp protease, ATP-binding subunit ClpX | | -2.43 | (0.70) |
| **PGN_1451** | *GroES* | chaperonin GroES | | +7.93 | (0.94) |
| **PGN_1715** | *GrpE* | putative chaperone protein GrpE | | +2.95 | (0.50) |
| **PG_1060** |  | carboxyl-terminal protease | | +8.08 | (4.41) |
| **PGN_1914** |  | carboxyl-terminal processing protease | | +5.99 | (0.39) |
| **PGN_0952** |  | carboxyl-terminal processing protease | | +5.53 | (1.84) |
| **PG_0088** |  | peptidase, M16 family | | -2.87 | (0.24) |
| **PGN_2035** |  | putative peptidase | | -2.74 | (0.55) |
| **PGN_1103** |  | Dipeptidase | | +8.89 | (2.53) |
| **PGN_0788** |  | peptidyl-dipeptidase | | +2.35 | (0.26) |
| **PG_1313** |  | dipeptidase-related protein | | +9.78 | (4.24) |
| **PG_1597** |  | DnaK suppressor protein, putative | | +2.32 | (0.19) |
| **PG_0256** |  | CvpA family protein | | -3.60 | (0.49) |
| **GENES RELATED TO AEROTOLERANCE** | | | | | |
| **PGN_0525** | *BatE* | probable aerotolerance-related exported protein BatE | | -8.28 | (0.63) |
| **PGN_0526** | *BatD* | aerotolerance-related exported protein BatD | | -4.74 | (1.14) |
| **PGN_0527** | *BatC* | probable aerotolerance-related exported protein BatC | | -5.25 | (0.65) |
| **PGN_0528** | *BatB* | putative aerotolerance-related exported protein BatB | | -5.15 | (1.70) |
| **PGN_0529** | *BatA* | aerotolerance-related membrane protein BatA | | -5.86 | (1.09) |
| **GENES RELATED TO CELL ENVELOPE** | | | | | |
| **PG_0180** |  | lipoprotein, putative | | -3.44 | (0.56) |
| **PG_0399** |  | lipoprotein, putative | | -2.34 | (0.29) |
| **PG_0679** |  | outer membrane efflux protein | | +2.27 | (0.08) |
| **PG_0922** |  | membrane protein, putative | | -7.29 | (2.61) |
| **PG_0924** |  | 5'-nucleotidase, lipoprotein e(P4) family | | -5.03 | (0.80) |
| **PG_1039** |  | integral membrane protein | | +2.41 | (0.30) |
| **PG_1180** |  | membrane protein, putative | | -2.48 | (0.59) |
| **PG_1587** |  | PAP2 superfamily protein | | -8.08 | (2.56) |
| **PG_1711** |  | alpha-1,2-mannosidase family protein | | -7.43 | (1.60) |
| **PG_1712** |  | alpha-1,2-mannosidase family protein | | -5.53 | (1.34) |
| **PG_1767** |  | lipoprotein, putative | | +2.85 | (0.57) |
| **PG_1828** |  | lipoprotein, putative | | +4.57 | (0.29) |
| **PG_2105** |  | lipoprotein, putative | | +3.08 | (0.25) |
| **PG_2133** |  | lipoprotein, putative | | -14.42 | (3.64) |
| **PG_2224** |  | membrane protein, putative | | -5.33 | (0.13) |
| **PGN_0081** |  | putative Na driven multidrug efflux pump | | -2.36 | (0.18) |
| **PGN_0405** |  | alpha-1,2-mannosidase family protein | | -5.85 | (0.31) |
| **PGN_1020** |  | probable ATP/GTP-binding transmembrane protein | | -4.36 | (0.83) |
| **GENES RELATED TO QUORUM SENSING** | | | | | |
| **PGN_1827** | *MetK* | S-adenosylmethionine synthase | | +2.55 | (0.26) |
| **GENES RELATED TO TRANSPORT** | | | | | |
|  | *MgtE* | magnesium transporter | | -2.24 | (0.27) |
| **PG_0091** |  | transporter, putative | | +2.87 | (0.71) |
| **PG_0321** |  | LAO/AO transport system ATPase | | -3.00 | (0.13) |
| **PG_0646** |  | iron compound ABC transporter, ATP-binding protein | | -3.16 | (0.62) |
| **PG_0647** |  | iron compound ABC transporter, permease protein | | -4.43 | (0.27) |
| **PG_0648** |  | iron compound ABC transporter, periplasmic iron compound-binding protein, putative | | -2.42 | (0.34) |
| **PG_0912** |  | polysaccharide transport protein, putative | | -2.36 | (0.05) |
| **PG_1010** |  | ABC transporter, ATP-binding protein | | -2.57 | (0.23) |
| **PGN_0685** |  | putative iron compound ABC transporter ATP-binding protein | | -2.67 | (0.05) |
| **PGN_0686** |  | putative iron compound ABC transporter permease protein | | -4.35 | (1.44) |
| **PGN_1343** |  | probable ABC transporter ATP-binding protein | | -2.81 | (0.71) |
| **PGN_1641** |  | arginine/ornithine transport system ATPase | | -3.77 | (0.06) |
| **PGN_1734** | *NupG* | nucleoside permease NupG | | -3.09 | (0.43) |
| **PGN_1876** |  | putative ABC transporter membrane protein | | -2.41 | (0.18) |
| **PGN_1898** |  | probable transport protein | | -2.93 | (0.49) |
| **GENES RELATED TO IRON METABOLISM** | | | | | |
|  | *HmuY* | hmuY protein | | +3.29 | (0.49) |
| **PG_0668** |  | TonB-dependent receptor | | -2.27 | (0.17) |
| **PG_1813** |  | ferredoxin, 4Fe-4S | | +2.25 | (0.24) |
| **PGN_0604** |  | Ferritin | | +7.28 | (1.54) |
| **PGN_0741** |  | TonB-dependent receptor | | +2.41 | (0.38) |
| **PGN_1058** | *Ftn* | Ferritin | | +6.11 | (1.48) |
| **GENES RELATED TO TRANSPOSON FUNCTIONS** | | | | | |
| **PG_1061** |  | ISPg6, transposase | | -2.71 | (0.91) |
| **PGN_0056** |  | probable conserved protein found in conjugate transposon | | +3.76 | (0.54) |
| **PGN_0057** | *TraP* | probable conserved protein found in conjugate transposon TraP. | | +3.99 | (0.70) |
| **PGN_0058** |  | probable conserved protein found in conjugate transposon | | +5.26 | (0.67) |
| **PGN_0059** | *TraN* | conserved protein found in conjugate transposon TraN | | +2.54 | (0.36) |
| **PGN_0060** | *TraM* | putative conserved protein found in conjugate transposon TraM | | +2.82 | (0.52) |
| **PGN_0062** | *TraK* | putative conserved protein found in conjugate transposon TraK | | +2.94 | (0.58) |
| **PGN_0063** | *TraJ* | conserved transmembrane protein found in conjugate transposon TraJ | | +2.68 | (0.63) |
| **PGN_0064** | *TraI* | putative conserved protein found in conjugate transposon TraI | | +3.11 | (1.07) |
| **PGN_0065** | *TraG* | conserved protein found in conjugate transposon TraG | | +3.49 | (0.39) |
| **PGN_0066** | *TraF* | probable conserved transmembrane protein found in conjugate transposon TraE | | +2.31 | (0.10) |
| **PGN_0069** | *TraA* | probable conserved protein found in conjugate transposon TraA | | +3.01 | (1.04) |
| **PGN_0592** | *TraQ* | putative conserved protein found in conjugate transposon TraQ | | +3.02 | (0.84) |
| **PGN_0954** |  | partial transposase in ISPg6 | | -3.33 | (0.40) |
| **PGN_1283** | *TraO* | conserved protein found in conjugate transposon TraO | | +5.20 | (0.20) |
| **PGN_1912** |  | partial transposase in ISPg6 | | -3.42 | (0.40) |
| **GENES RELATED TO CRISPR** | | | | | |
|  | *Cas2-1* | CRISPR-associated protein Cas2 | | -2.93 | (0.81) |
| **PGN_1959** | *Cas2-2* | CRISPR-associated protein Cas2 | | +4.80 | (0.50) |
| **GENES RELATED TO RIBOSOME/ RNA** | | | | | |
|  | *RpmI* | probable 50S ribosomal protein L35 | | +2.87 | (0.68) |
|  | *RnpA* | ribonuclease P protein component | | -2.66 | (0.27) |
|  | *RbfA* | ribosome-binding factor A | | -7.81 | (1.48) |
|  | *PyrC* | Dihydroorotase | | -2.81 | (0.53) |
|  | *KsgA* | dimethyladenosine transferase | | -2.53 | (0.24) |
| **PG_0075** |  | phosphoribosylformylglycinamidine cyclo-ligase, putative | | -2.33 | (0.28) |
| **PG_0627** |  | RNA-binding protein | | +2.47 | (0.05) |
| **PGN_0167** | *RpsP* | 30S ribosomal protein S16 | | +2.38 | (0.25) |
| **PGN_0188** | *RpmF* | 50S ribosomal protein L32 | | +2.66 | (0.36) |
| **PGN_0394** | *RpsT* | probable 30S ribosomal protein S20 | | +2.72 | (0.41) |
| **PGN_0668** |  | RNA-binding protein | | +2.42 | (0.17) |
| **PGN_0694** | *RpmH* | 50S ribosomal protein L34 | | +4.37 | (0.59) |
| **PGN_0761** |  | ribosomal large subunit pseudouridine synthase | | -3.11 | (0.26) |
| **PGN_0965** | *RplT* | putative 50S ribosomal protein L20 | | +2.94 | (0.56) |
| **PGN_1024** |  | putative ribosome-binding factor A | | -6.88 | (1.16) |
| **PGN_1088** | *RpsA* | 30S ribosomal protein S1 | | +2.80 | (0.18) |
| **PGN_1840** | *RplQ* | 50S ribosomal protein L17 | | +2.62 | (0.38) |
| **PGN_1871** | *RpsG* | 30S ribosomal protein S7 | | -2.57 | (0.41) |
| **GENES RELATED TO FIMBRIA** | | | | | |
| **PGN_0180** | *FimA* | FimA type I fimbrilin | | -11.03 | (1.71) |
| **PGN_0181** |  | Fimbrillin-A associated anchor proteins Mfa1 and Mfa2 | | -8.41 | (2.84) |
| **PGN_0183** | *FimC* | minor component FimC | | -17.73 | (2.28) |
| **PGN_0184** | *FimD* | minor component FimD | | -9.65 | (1.85) |
| **GENES RELATED TO TRANSCRIPTION AND TRANSLATION** | | | | | |
|  | *NrdG* | anaerobic ribonucleoside-triphosphate reductase activating protein | | -8.42 | (1.42) |
| **PG_0020** |  | transcriptional regulator, MarR family | | +2.43 | (0.52) |
| **PG_0997** |  | transcriptional regulator, putative | | +2.50 | (0.31) |
| **PG_1260** |  | anaerobic ribonucleoside-triphosphate reductase, putative | | -12.50 | (4.82) |
| **PG_2000** |  | signal peptidase-related protein | | -2.45 | (0.35) |
| **PGN_0082** |  | probable transcriptional regulator AraC family | | -2.99 | (0.59) |
| **PGN_0319** |  | probable RNA polymerase sigma-70 factor ECF subfamily | | +2.66 | (0.23) |
| **PGN_0355** |  | translation initiation factor IF-2 | | -3.00 | (0.41) |
| **PGN_0782** |  | putative tRNA pseudouridine synthase A | | +2.70 | (0.52) |
| **PGN_0945** |  | putative TetR family transcriptional regulator | | -2.36 | (0.08) |
| **PGN_0970** |  | putative RNA polymerase sigma-70 factor ECF subfamily | | +4.06 | (0.70) |
| **PGN_1226** |  | ribonucleotide reductase | | -2.91 | (0.52) |
| **PGN_1312** |  | probable transcriptional regulator as Arg-repressor | | +2.32 | (0.05) |
| **PGN_1395** |  | putative anaerobic ribonucleoside-triphosphate reductase activating protein | | -5.47 | (0.52) |
| **PGN_1396** |  | anaerobic ribonucleoside-triphosphate reductase | | -14.51 | (2.00) |
| **GENES RELATTED TO FATTY ACID AND PHOSPOLIPID METABOLISM** | | | | | |
| **PG_1155** |  | ADP-heptose--LPS heptosyltransferase, putative | | -6.31 | (1.56) |
| **PGN_1255** | *Rfa* | putative heptosyltransferase | | -4.81 | (2.33) |
| **GENES RELATED TO DNA METABOLISM** | | | | | |
|  | *XseA* | exodeoxyribonuclease VII, large subunit | | -2.60 | (0.37) |
|  | *Tmk* | thymidine kinase | | -2.67 | (0.56) |
|  | *RecJ* | single-stranded-DNA-specific exonuclease RecJ | | -2.92 | (0.23) |
|  | *PurB* | adenylosuccinate lyase | | +2.53 | (0.37) |
|  | *GuaB* | inosine-5'-monophosphate dehydrogenase | | +2.80 | (0.68) |
| **PG_0030** |  | cytidine deaminase | | +3.77 | (0.32) |
| **PG_0174** |  | pyridine nucleotide-disulphide oxidoreductase family protein | | -2.72 | (0.43) |
| **PG_1038** |  | ATP-dependent DNA helicase UvrD/PcrA/Rep Family | | -9.02 | (4.61) |
| **PGN_0001** | *DnaA* | chromosomal replication initiator protein DnaA | | +2.12 | (0.08) |
| **PGN_0026** |  | putative cytidine deaminase | | +4.05 | (0.41) |
| **PGN_0084** |  | DNA topoisomerase I | | +6.92 | (0.11) |
| **PGN_0923** |  | putative DNA primase | | -2.62 | (0.25) |
| **PGN_1022** |  | putative thymidine kinase | | -3.94 | (0.40) |
| **PGN_1027** |  | Dihydroorotase | | -3.26 | (0.72) |
| **PGN_1225** |  | probable exodeoxyribonuclease VII large subunit | | -2.91 | (0.25) |
| **PGN_1314** |  | ATP-dependent DNA helicase | | -8.90 | (4.14) |
| **PGN_1449** |  | inosine-5'-monophosphate dehydrogenase | | +3.36 | (0.34) |
| **PGN_1567** | *RecF* | recF protein | | -2.69 | (0.33) |
| **PGN_1992** |  | putative helicase | | -2.34 | (0.08) |
| **GENES RELATED TO ENERGY METABOLISM** | | | | | |
|  | *PckA* | phosphoenolpyruvate carboxykinase (ATP) | | +2.27 | (0.22) |
|  | *HprA* | glycerate dehydrogenase | | +2.54 | (0.32) |
| **PG_1834** |  | glycogen synthase-related protein | | -3.24 | (1.07) |
| **PG_2171** |  | D-isomer specific 2-hydroxyacid dehydrogenase family protein | | -2.36 | (0.23) |
| **PG_2213** |  | nitrite reductase-related protein | | -2.76 | (0.18) |
| **PGN_1120** |  | putative NADPH-NAD transhydrogenase | | +3.08 | (1.09) |
| **PGN_1736** |  | putative glycogen synthase | | -3.51 | (0.31) |
| **PGN_1746** | *NrfA* | cytochrome c nitrite reductase, catalytic subunit NrfA | | -2.92 | (0.64) |
| **GENES RELATED TO DNA BINDING PROTEINS** | | | | | |
|  | *Hup-2* | DNA-binding protein HU | | +2.85 | (0.46) |
| **PG_0254** |  | N utilization substance protein A, putative | | -4.15 | (0.89) |
| **PG_0555** |  | DNA-binding protein, histone-like family | | +3.76 | (0.50) |
| **PG_2040** |  | DNA-binding protein, histone-like family | | +4.06 | (0.85) |
| **PGN_1393** |  | putative DNA-binding protein HU | | +2.97 | (0.80) |
| **PGN_1415** |  | DNA-binding protein histone-like family | | +3.90 | (0.42) |
| **PGN_1986** |  | DNA-binding protein, histone-like family | | +4.28 | (0.61) |
| **GENES RELATED TO OTHER FUNCTIONS** | | | | | |
|  | *LysC* | aspartate kinase | | -2.59 | (0.25) |
|  | *LysA* | diaminopimelate decarboxylase | | -2.46 | (0.32) |
|  | *KdsA* | 2-dehydro-3-deoxyphosphooctonate aldolase | | +2.44 | (0.32) |
|  | *FolP* | dihydropteroate synthase | | +2.35 | (0.21) |
|  | *Dxr* | 1-deoxy-D-xylulose 5-phosphate reductoisomerase | | -4.08 | (0.32) |
| **PG_0079** |  | abortive infection protein, putative | | +4.64 | (1.48) |
| **PG_0199** |  | TatD family protein | | -3.78 | (0.11) |
| **PG_0226** |  | transglutaminase-related protein | | +2.41 | (0.29) |
| **PG_0917** |  | GtrA family protein | | -3.25 | (0.60) |
| **PG_0920** |  | glycosyl transferase, group 2 family protein | | -3.63 | (1.90) |
| **PG_1014** |  | TPR domain protein | | -2.58 | (0.37) |
| **PG_1840** |  | conserved domain protein | | -3.63 | (0.47) |
| **PG_1963** |  | Sua5/YciO/YrdC/YwlC family protein | | +2.20 | (0.11) |
| **PG_2028** |  | ebsC protein | | -2.62 | (0.47) |
| **PG_2131** |  | 60 kDa protein/OmpA_C-like | | -19.57 | (6.37) |
| **PGN_0082** | *AroC* | chorismate synthase | | -3.39 | (0.73) |
| **PGN_0094** |  | putative bacteriophage integrase | | +2.78 | (0.57) |
| **PGN_0101** |  | putative 1,4-dihydroxy-2-naphthoate octaprenyltransferase | | -3.50 | (0.69) |
| **PGN_0179** |  | 60 kDa protein | | -17.32 | (2.32) |
| **PGN_0232** |  | probable glycosyl transferase family 2 | | -2.52 | (0.43) |
| **PGN_0243** |  | phosphoglycerate mutase | | +2.50 | (0.19) |
| **PGN_0248** |  | putative dimethyladenosine transferase | | -2.71 | (0.24) |
| **PGN_0285** |  | pyridine nucleotide-disulphide oxidoreductase | | -2.71 | (0.60) |
| **PGN_0354** |  | putative nitrogen utilization substance protein A | | -4.30 | (0.98) |
| **PGN_0406** |  | conserved hypothetical protein with glycosyl hydrolase family 92 domain | | -6.40 | (2.32) |
| **PGN_0466** |  | putative cardiolipin synthetase | | +2.30 | (0.13) |
| **PGN_0518** |  | putative ribulose-phosphate 3-epimerase | | +2.94 | (0.52) |
| **PGN_0522** |  | putative dihydropteroate synthase | | +2.51 | (0.16) |
| **PGN_0524** |  | lipid A 4'-phosphatase | | -8.40 | (1.72) |
| **PGN_0531** |  | putative von Willebrand factor type A | | -3.09 | (0.77) |
| **PGN_0571** |  | putative undecaprenol kinase | | -3.25 | (0.11) |
| **PGN_0743** |  | probable FKBP-type peptidyl-prolyl cis-trans isomerase FkpA | | +2.50 | (0.29) |
| **PGN_0753** |  | probable two component system response regulator | | +2.87 | (0.33) |
| **PGN_0917** |  | tyrosine type site-specific recombinase | | -4.39 | (1.10) |
| **PGN_0975** | *MenA* | 1,4-dihydroxy-2-naphthoate octaprenyltransferase | | -3.11 | (0.31) |
| **PGN_1023** |  | acid phosphatase OlpA | | -5.16 | (1.03) |
| **PGN_1089** |  | probable methyltransferase | | -4.30 | (0.85) |
| **PGN_1104** |  | chorismate synthase | | -3.68 | (0.50) |
| **PGN_1151** |  | 1-deoxy-D-xylulose-5-phosphate reductoisomerase | | -3.93 | (0.87) |
| **PGN_1206** |  | putative methylenetetrahydrofolate dehydrogenase | | +3.08 | (0.68) |
| **PGN_1209** |  | probable flavodoxin | | +2.82 | (0.20) |
| **PGN_1220** |  | adenylosuccinate lyase | | +2.53 | (0.42) |
| **PGN_1221** |  | probable ATP:corrinoid adenosyltransferase | | +3.98 | (1.69) |
| **PGN_1272** |  | putative diaminopimelate decarboxylase | | -2.53 | (0.24) |
| **PGN_1273** |  | probable 1,4-dihydroxy-2-naphthoate octaprenyltransferase | | -3.23 | (0.67) |
| **PGN_1706** |  | probable phosphoribosylglycinamide formyltransferase | | +3.37 | (0.53) |
| **PGN_1748** |  | putative cytochrome c biogenesis protein CcsA | | -2.82 | (0.44) |
| **PGN_1975** |  | putative regulatory protein | | -2.59 | (0.60) |
| **PGN_1985** |  | probable N-acetylmuramoyl-L-alanine amidase | | +3.52 | (0.11) |
| **PGN_2026** |  | putative abortive infection protein | | +4.40 | (0.71) |
| **GENES RELATED TO HYPOTHETICAL PROTEIN** | | | | | |
| **PG_0031** |  | hypothetical protein | | +3.28 | (0.22) |
| **PG_0039** |  | hypothetical protein | | -2.24 | (0.10) |
| **PG_0161** |  | hypothetical protein | | +2.74 | (0.39) |
| **PG_0164** |  | conserved hypothetical protein | | -3.24 | (0.68) |
| **PG_0179** |  | hypothetical protein | | -3.48 | (1.00) |
| **PG_0204** |  | hypothetical protein | | +2.47 | (0.30) |
| **PG_0229** |  | hypothetical protein | | -2.69 | (0.42) |
| **PG_0286** |  | hypothetical protein | | +3.57 | (0.53) |
| **PG_0323** |  | conserved hypothetical protein | | +3.10 | (0.02) |
| **PG_0404** |  | hypothetical protein | | +4.53 | (0.48) |
| **PG_0409** |  | hypothetical protein | | +2.24 | (0.09) |
| **PG_0421** |  | hypothetical protein | | +5.43 | (1.62) |
| **PG_0447** |  | conserved hypothetical protein | | +2.99 | (0.27) |
| **PG_0448** |  | hypothetical protein | | +3.08 | (0.34) |
| **PG_0547** |  | conserved hypothetical protein | | -2.71 | (0.41) |
| **PG_0612** |  | hypothetical protein | | -2.60 | (0.21) |
| **PG_0613** |  | hypothetical protein | | -2.83 | (0.52) |
| **PG_0883** |  | hypothetical protein | | -2.52 | (0.75) |
| **PG_0898** |  | conserved hypothetical protein | | +2.56 | (0.27) |
| **PG_0918** |  | hypothetical protein | | -2.89 | (0.64) |
| **PG_0926** |  | hypothetical protein | | -4.78 | (0.59) |
| **PG_0927** |  | conserved hypothetical protein TIGR00150 | | -5.33 | (0.60) |
| **PG_0929** |  | hypothetical protein | | +2.48 | (0.32) |
| **PG_0994** |  | hypothetical protein | | -3.70 | (1.33) |
| **PG_1085** |  | hypothetical protein | | +2.96 | (0.40) |
| **PG_1257** |  | hypothetical protein | | +3.03 | (0.24) |
| **PG_1300** |  | conserved hypothetical protein | | -2.99 | (0.93) |
| **PG_1388** |  | hypothetical protein | | -2.65 | (0.27) |
| **PG_1398** |  | hypothetical protein | | -2.05 | (0.05) |
| **PG_1546** |  | hypothetical protein | | +3.91 | (0.56) |
| **PG_1661** |  | hypothetical protein | | -2.50 | (0.32) |
| **PG_1715** |  | hypothetical protein | | -3.66 | (0.31) |
| **PG_1817** |  | conserved hypothetical protein | | -3.20 | (1.17) |
| **PG_1818** |  | hypothetical protein | | -2.63 | (0.37) |
| **PG_1819** |  | hypothetical protein | | -4.50 | (0.89) |
| **PG_1908** |  | hypothetical protein | | -3.20 | (0.83) |
| **PG_1966** |  | conserved hypothetical protein | | -3.24 | (0.59) |
| **PG_1997** |  | hypothetical protein | | +2.41 | (0.32) |
| **PG_2031** |  | hypothetical protein | | -2.79 | (0.24) |
| **PG_2037** |  | hypothetical protein | | +2.53 | (0.71) |
| **PG_2101** |  | hypothetical protein | | -8.79 | (2.11) |
| **PG_2106** |  | hypothetical protein | | +3.07 | (0.66) |
| **PG_2130** |  | hypothetical protein | | -17.17 | (3.46) |
| **PG_2139** |  | hypothetical protein | | +2.86 | (0.18) |
| **PG_2204** |  | hypothetical protein | | +3.88 | (0.36) |
| **PG_2212** |  | hypothetical protein | | -7.57 | (0.89) |
| **PG_2225** |  | conserved hypothetical protein | | -3.22 | (0.49) |
| **PG_2226** |  | hypothetical protein | | -3.50 | (0.68) |
| **PGN_0029** |  | conserved hypothetical protein | | +2.74 | (0.35) |
| **PGN_0053** |  | conserved hypothetical protein | | +2.31 | (0.19) |
| **PGN_0061** |  | hypothetical protein | | +3.42 | (0.79) |
| **PGN_0068** |  | hypothetical protein | | +2.99 | (0.16) |
| **PGN_0070** |  | hypothetical protein | | +2.37 | (0.05) |
| **PGN_0072** |  | hypothetical protein | | +3.77 | (1.28) |
| **PGN_0083** |  | conserved hypothetical protein | | +4.85 | (0.80) |
| **PGN_0090** |  | hypothetical protein | | +2.71 | (0.52) |
| **PGN_0091** |  | hypothetical protein | | +3.78 | (1.17) |
| **PGN_0092** |  | conserved hypothetical protein | | +3.40 | (0.10) |
| **PGN_0093** |  | conserved hypothetical protein | | +5.14 | (0.24) |
| **PGN_0110** |  | hypothetical protein | | +2.73 | (0.21) |
| **PGN_0124** |  | hypothetical protein | | -3.68 | (0.57) |
| **PGN_0154** |  | conserved hypothetical protein | | +3.66 | (0.07) |
| **PGN_0156** |  | conserved hypothetical protein | | +2.97 | (0.36) |
| **PGN_0164** |  | conserved hypothetical protein | | -3.69 | (0.26) |
| **PGN_0178** |  | conserved hypothetical protein | | -16.48 | (1.52) |
| **PGN_0182** |  | conserved hypothetical protein | | -16.16 | (2.92) |
| **PGN_0187** |  | conserved hypothetical protein | | +2.85 | (0.68) |
| **PGN_0273** |  | conserved hypothetical protein | | +3.09 | (0.32) |
| **PGN_0288** |  | conserved hypothetical protein | | -3.09 | (1.08) |
| **PGN_0289** |  | conserved hypothetical protein | | -4.02 | (0.76) |
| **PGN_0307** |  | conserved hypothetical protein | | -3.45 | (0.70) |
| **PGN_0312** |  | hypothetical protein | | +2.39 | (0.29) |
| **PGN_0332** |  | conserved hypothetical protein | | -2.42 | (0.40) |
| **PGN_0356** |  | conserved hypothetical protein | | -3.79 | (0.60) |
| **PGN_0400** |  | conserved hypothetical protein | | -5.05 | (1.30) |
| **PGN_0404** |  | conserved hypothetical protein | | -3.56 | (0.33) |
| **PGN_0481** |  | hypothetical protein | | -4.01 | (1.08) |
| **PGN_0511** |  | conserved hypothetical protein | | +2.42 | (0.11) |
| **PGN_0530** |  | conserved hypothetical protein | | -4.40 | (0.41) |
| **PGN_0538** |  | conserved hypothetical protein | | -2.72 | (0.11) |
| **PGN_0562** |  | conserved hypothetical protein | | +2.53 | (0.35) |
| **PGN_0578** |  | conserved hypothetical protein found in conjugate transposon | | +2.39 | (0.23) |
| **PGN_0583** |  | conserved hypothetical protein | | -2.52 | (0.39) |
| **PGN_0586** |  | conserved hypothetical protein | | -2.63 | (0.48) |
| **PGN_0589** |  | conserved hypothetical protein | | -2.67 | (0.08) |
| **PGN_0654** |  | conserved hypothetical protein | | -2.50 | (0.36) |
| **PGN_0655** |  | conserved hypothetical protein | | -2.56 | (0.27) |
| **PGN_0656** |  | conserved hypothetical protein | | -2.67 | (0.40) |
| **PGN_0752** |  | hypothetical protein | | +3.92 | (0.35) |
| **PGN_0794** |  | conserved hypothetical protein | | -2.42 | (0.26) |
| **PGN_0797** |  | conserved hypothetical protein | | +2.53 | (0.09) |
| **PGN_0835** |  | conserved hypothetical protein | | -3.19 | (0.57) |
| **PGN_0874** |  | conserved hypothetical protein | | -2.98 | (0.33) |
| **PGN_0978** |  | hypothetical protein | | -2.59 | (0.34) |
| **PGN_1017** |  | conserved hypothetical protein | | -3.99 | (0.36) |
| **PGN_1021** |  | hypothetical protein | | -5.92 | (0.75) |
| **PGN_1025** |  | conserved hypothetical protein | | -8.12 | (2.36) |
| **PGN_1028** |  | conserved hypothetical protein | | -2.96 | (0.20) |
| **PGN_1029** |  | conserved hypothetical protein | | -3.28 | (0.36) |
| **PGN_1081** |  | conserved hypothetical protein | | +2.50 | (0.33) |
| **PGN_1159** |  | conserved hypothetical protein | | -2.35 | (0.21) |
| **PGN_1182** |  | conserved hypothetical protein | | +2.84 | (0.27) |
| **PGN_1254** |  | hypothetical protein | | -2.46 | (0.37) |
| **PGN_1256** |  | conserved hypothetical protein | | -3.86 | (0.26) |
| **PGN_1306** |  | hypothetical protein | | -5.89 | (2.38) |
| **PGN_1307** |  | hypothetical protein | | -8.18 | (1.61) |
| **PGN_1313** |  | conserved hypothetical protein | | +3.23 | (0.49) |
| **PGN_1337** |  | conserved hypothetical protein | | -2.37 | (0.21) |
| **PGN_1340** |  | conserved hypothetical protein | | -2.62 | (0.15) |
| **PGN_1380** |  | hypothetical protein | | +3.53 | (0.63) |
| **PGN_1385** |  | hypothetical protein | | +3.63 | (0.48) |
| **PGN_1392** |  | conserved hypothetical protein | | +2.89 | (0.50) |
| **PGN_1419** |  | conserved hypothetical protein | | -2.65 | (0.68) |
| **PGN_1435** |  | hypothetical protein | | +2.30 | (0.18) |
| **PGN_1436** |  | conserved hypothetical protein | | +2.66 | (0.43) |
| **PGN_1438** |  | hypothetical protein | | -2.17 | (0.13) |
| **PGN_1476** |  | conserved hypothetical protein | | +3.53 | (0.09) |
| **PGN_1496** |  | hypothetical protein | | +3.77 | (0.56) |
| **PGN_1509** |  | conserved hypothetical protein | | +2.26 | (0.25) |
| **PGN_1515** |  | conserved hypothetical protein | | +2.65 | (0.33) |
| **PGN_1547** |  | conserved hypothetical protein | | +6.85 | (1.74) |
| **PGN_1561** |  | conserved hypothetical protein | | +5.07 | (1.10) |
| **PGN_1591** |  | conserved hypothetical protein | | +2.38 | (0.49) |
| **PGN_1609** |  | hypothetical protein | | +2.46 | (0.28) |
| **PGN_1639** |  | conserved hypothetical protein | | +3.43 | (0.49) |
| **PGN_1661** |  | conserved hypothetical protein | | +2.44 | (0.23) |
| **PGN_1678** |  | conserved hypothetical protein | | +3.55 | (0.35) |
| **PGN_1731** |  | conserved hypothetical protein | | -3.23 | (0.87) |
| **PGN_1739** |  | conserved hypothetical protein | | +3.97 | (0.61) |
| **PGN_1747** |  | conserved hypothetical protein | | -3.05 | (0.58) |
| **PGN_1837** |  | conserved hypothetical protein | | -2.90 | (0.20) |
| **PGN_1878** |  | conserved hypothetical protein | | +2.62 | (0.37) |
| **PGN_1889** |  | conserved hypothetical protein | | +2.36 | (0.19) |
| **PGN_1920** |  | conserved hypothetical protein | | -2.40 | (0.06) |
| **PGN_1923** |  | hypothetical protein | | -3.06 | (0.68) |
| **PGN_1924** |  | conserved hypothetical protein | | -2.85 | (0.57) |
| **PGN_1942** |  | hypothetical protein | | +2.24 | (0.07) |
| **PGN_1965** |  | hypothetical protein | | -2.64 | (0.30) |
| **PGN_1966** |  | conserved hypothetical protein | | -2.70 | (0.54) |
| **PGN_1984** |  | hypothetical protein | | +2.28 | (0.22) |
| **PGN_2000** |  | hypothetical protein | | -2.32 | (0.21) |
| **PGN_2004** |  | conserved hypothetical protein | | -2.46 | (0.17) |
| **PGN_2038** |  | conserved hypothetical protein | | +3.54 | (0.37) |
| **PGN_2070** |  | conserved hypothetical protein | | +3.29 | (0.32) |
| **PGN_2076** |  | conserved hypothetical protein | | -8.19 | (0.74) |
| **PGN_2088** |  | conserved hypothetical protein | | -5.95 | (0.59) |
| **PGN_2089** |  | conserved hypothetical protein | | -3.46 | (0.65) |
| **PGN_2090** |  | conserved hypothetical protein | | -3.35 | (0.63) |
